# The non‐coding RNome after splenectomy

**DOI:** 10.1111/jcmm.14664

**Published:** 2019-09-08

**Authors:** Mihnea P. Dragomir, Stefan Tudor, Keishi Okubo, Masayoshi Shimizu, Meng Chen, Dana Elena Giza, William Ruixian He, Cristina Ivan, George A. Calin, Catalin Vasilescu

**Affiliations:** ^1^ Department of Experimental Therapeutics The University of Texas MD Anderson Cancer Center Houston TX USA; ^2^ Department of Surgery Fundeni Clinical Hospital Carol Davila University of Medicine and Pharmacy Bucharest Romania; ^3^ Department of Family and Community Medicine McGovern Medical School at The University of Texas Health Science Center at Houston Houston TX USA; ^4^ Center for RNA Interference and Non‐coding RNAs The University of Texas MD Anderson Cancer Center Houston TX USA

**Keywords:** microRNA, non‐coding RNA, OPSI, pyknon, sepsis, splenectomy

## Abstract

Splenectomy is a common surgical procedure performed in millions of people worldwide. Epidemiologic data show that splenectomy is followed by infectious (sepsis) and non‐infectious complications, with unknown mechanisms. In order to explore the role of the non‐coding transcripts involved in these complications, we analysed a panel of circulating microRNAs (miRNAs), which were previously reported to be deregulated in sepsis, in the plasma of splenectomized patients. MiR‐223 was overexpressed immediately and late after splenectomy, while miR‐146a was overexpressed immediately after splenectomy, returning latter to basal levels; and miR‐16, miR‐93, miR‐26a and miR‐26b were overexpressed only late after splenectomy, suggesting similarities with sepsis. We also explored the non‐coding (nc)RNome of circulating peripheral blood leucocytes by performing a ncRNA full genome profiling. We observed a reorganization of the ncRNoma after splenectomy, characterized by up‐regulation of miRNAs and down‐regulation of transcribed pyknons (T‐PYKs). Pathway analysis revealed that deregulated miRNAs control pathways involved in immunity, cancer and endothelial growth. We checked the expression of the ncRNAs in 15 immune cell types from healthy donors and observed that plasma miRNAs, cellular miRNAs and T‐PYKs have a cell‐specific expression pattern and are abundant in different types of immune cells. These findings suggest that the ncRNAs potentially regulate the immune changes observed after splenectomy.

## 1 INTRODUCTION

The cause of asplenia or hyposplenism can be congenital, functional or surgical.^1^ Splenectomy is a common elective surgical procedure for different haematological conditions: hereditary spherocytosis, immune thrombocytopenic purpura, sickle cell anaemia, myelofibrosis and also a part of more complex surgical approaches for malignant diseases where en bloc resection of the spleen is necessary.^2^ Additionally, splenectomy is performed as an emergency surgery for trauma where the rupture of the organ needs to be quickly addressed.^3^ In the United States, there are over 1 million asplenic patients and 25 000 new splenectomies are performed yearly.^1^ The spleen was regarded for decades as a dispensable organ, and its removal would not affect the morbidity and mortality of the patients.^4^ Only recently, numerous infectious and non‐infectious complications were linked to splenectomy.^5^ The spleen is an organ abundant in innate immune cells, including macrophages essential for the elimination of encapsulated bacteria.^6^ Additionally, the spleen is the site where M immunoglobulins are produced by memory B cells, which have an important role in eradicating pathogens from blood.^7^, ^8^ Therefore, the most feared complication of splenectomy is sepsis, referred to as overwhelming post‐splenectomy infection (OPSI). The risk for sepsis is lifelong, and the infection has often a fulminant course.^9^

Despite intense research,^10^, ^11^ the exact mechanisms that predispose asplenic/hyposplenic patients to OPSI and other complications remain elusive. In the last years, because of these complications, and the development of novel non‐invasive therapies, elective splenectomy is avoided by haematologist despite known benefits and lesser economic impact.^12^

Herein, we propose a paradigm‐shifting approach in which we explore the role of various categories of non‐coding RNA (ncRNA) from plasma and immune cells of splenectomized patients in order to understand the non‐coding RNome deregulations induced by splenectomy. NcRNAs comprise a broad range of RNA species including, among many others, long non‐coding RNAs (lncRNAs),^13^ transcribed ultraconserved non‐coding RNAs (T‐UCRs),^14^, ^15^ microRNAs (miRNAs),^16^, ^17^ transcribed human/primate‐specific pyknons (T‐PYKs),^18^ small interfering RNAs (siRNAs)^19^ and piwi‐interacting RNAs (piRNAs).^20^ The ncRNome has been recently described as a regulator of the immune system,^21^, ^22^ but its deregulation after splenectomy has not been studied.

In our previous studies, we detected by genome‐wide screenings a panel of human and viral small non‐coding miRNAs that are deregulated in peripheral blood mono‐nuclear cells (PBMC) of septic patients.^23^ We confirmed by RT‐qPCR that several of these miRNAs are also deregulated in the plasma of septic patients.^24^, ^25^ Next, we built miRNA networks for septic patients and healthy volunteers using the expression of these miRNAs and observed that in sepsis the miRNA network is fragmented, containing less edges and more isolated nodes.^26^ Therefore, we hypothesized that the sepsis specific human and viral miRNAs could be deregulated in the circulating peripheral leucocytes and/or plasma of patients after splenectomy and could, at least in part, explain the predisposition towards infections in this patient group. We consider that (a) the ncRNA expression after splenectomy could be used to detect the splenectomized patients at risk for sepsis and (b) the splenectomized patients at risk for OPSI could improve our understanding of sepsis—a disease for which clinicians are still in search for a cure.^27^, ^28^

## 2 METHODS

### 2.1 Clinical samples

The study subjects (both for the plasma study subjects, n = 27, Table S1 and peripheral blood leucocytes array profiling, n = 11, Table S2) were recruited prospectively between 2013 and 2015 from Fundeni Clinical Hospital (FCH), Bucharest, Romania. All clinical data and blood samples were obtained from participants who had given written informed consent, according to protocols approved by the FCH Ethics Committee. Blood was sampled from patients prior to elective splenectomy (D0), 7 days (D7), 30 days (D30), 90 days (D90), 180 days (D180) and 360 days after splenectomy (D360). All blood samples were collected in 10 mL EDTA tubes, plasma and peripheral blood leucocytes were processed, frozen in aliquots, and stored at −80°C after collection. All patients included in the study were vaccinated against *Haemophilus influenzae*, *Neisseria meningitidis* and *Streptococcus pneumonia*.

### 2.2 RNA extraction from plasma/peripheral blood leucocytes and reverse transcription (RT)

Total RNA was extracted and reverse transcribed as previously described.^25^, ^29^ Briefly, RNA was obtained from 100 µL of plasma or peripheral blood leucocytes using the total RNA purification kit (NorgenBiotek, Cat. #37500) according to the manufacturer's protocol. RNA was eluted in 50 µL elution solution and RNA concentrations and quality were assessed using NanoDrop‐1100. For the normalization of plasma sample‐to‐sample variation in the RNA isolation step, the *Caenorhabditis elegans,* cel‐miR‐39‐3p and cel‐miR‐54‐3p, (ThermoFisher SCIENTIFIC, Cat # A25576 and Cat #A25576), 25 fmol of each in a total volume of 1 μL, were used. For the normalization of sample‐to‐sample variation of RNA extracted from peripheral blood leucocytes, U6 was used as an endogenous normalizer. RNA was reverse transcribed using the TaqMan^®^ miRNA Reverse Kit (Applied Biosystems, Cat. #4366596) in 10 μL RT reaction containing 10 ng of RNA, 0.1 μL of 100 mM dNTPs, 0.67 μL of Multiscribe reverse transcriptase, 1 μL of 10× RT buffer, 0.13 μL of RNase inhibitor and 1 μL of 5× miRNA‐specific stem‐loop RT primer (Applied Biosystems). Reverse transcription was performed in a Bio‐Rad DNA engine with the following program: 16°C for 30 minutes, 42°C for 30 minutes, 85°C for 5 minutes and then 4°C on hold. The cDNA was diluted and stored at −20°C until analysis.

### 2.3 Real‐Time RT‐qPCR profiling and normalization

The diluted cDNA (3 µL) was used as template in a quantitative PCR (qPCR) reaction with a total final volume of 5 µL. DNA amplification was performed using TaqMan primers/probes specific for each miRNA (plasma: the 12 miRNAs previously detected by microarray to be deregulated in sepsis^24^ and four additional miRNAs we used for the previously described sepsis miRNA network^26^; peripheral blood leucocytes: miR‐324 and miR‐335) together with SsoFast™ Probes Supermix (Bio‐Rad Laboratories, Cat. #172‐5231). The reaction started with incubation for 3 minutes at 95°C followed by 40 cycles of 5 seconds at 95°C and 30 seconds at 60°C. All experiments were performed in triplicate. Ct values beyond the upper limit of the measuring system are imputed as 35. The raw Ct values, for the plasma samples, were normalized by Ct values of cel‐miR‐54‐3p the exogenous normalizers (ΔCt = Ct gene – Ct cel‐miR‐54). We selected cel‐miR‐54‐3p as normalizer, because it proved to be the most stable normalization method between the groups for the final analysis (smallest SD, no expression value over 30 cycles and no statistical difference between groups) (Figure S1A). For peripheral blood leucocytes samples, we used U6 as endogenous control (ΔCt = Ct gene – Ct U6). U6 proved to be a stable normalization method between the groups for the final analysis (Figure S1B). Finally, the relative expression of each miRNA was calculated using the equation 2^−ΔCT^.

### 2.4 Array design and data analysis

The arrays utilize nucleic acid hybridization of a 52 nt biotin‐labelled cDNA target with DNA oligonucleotide probes attached to a gel matrix. The biotin‐labelled cDNA targets are prepared by a reverse transcription into first strand cDNA. Total RNA is primed for reverse transcription by a random octamer conjugated with two biotins and a 52 nt long poly‐A tail. This procedure results in an equal copy number of biotin cDNA targets to the ncRNA templates. The array contains a collection of probes for various types of ncRNAs: 18 009 probes corresponding to 1271 human pre‐miRNAs, 8660 probes corresponding to 626 mouse pre‐miRNAs (miRBase 21), 2745 probes corresponding to 479 ultraconserved elements, 16 314 probes corresponding to 1283 T‐PYKs and 2197 probes corresponding to 97 lncRNAs. Some of the probes are designed from upstream or downstream regions of certain ncRNAs.

The arrays were analysed in R (version 3.5.1) (http://www.r-project.org/). Data pre‐processing steps of background‐correction, normalization and summarization were performed using functions in Limma library (http://www.bioconductor.org/packages/devel/bioc/vignettes/limma/inst/doc/usersguide.pdf). A threshold for positive spot selection for microarray data was calculated as the mean value of all the dark corner spots plus twice the standard deviation.^30^ Differential expression of ncRNAs in a comparative analysis between pre‐ and post‐splenectomy cases was determined by a fold change in absolute value equal or greater to 1.1 and a *P*‐value obtained from the moderated t‐statistic from LIMMA package <.05. Heatmaps displaying the most differentiated genes were generated using the heatmap.2 function of gplots library. We performed two independent ncRNA array profiling of peripheral blood leucocytes: 2017 training and 2019 validation. The data were submitted to GEO and were assigned specific GEO accession numbers: GSE133588 for 2017 training cohort and GSE133589 for 2019 validation cohort.

### 2.5 miRNA target prediction and pathway analysis

For miRNAs, we obtained experimentally confirmed miRNA‐mRNA interactions from miRTarBase (http://mirtarbase.mbc.nctu.edu.tw). Both weak and strong experimentally validated target mRNAs were selected for integrated pathway analysis with KEGG, Wikipathways, Reactome, BioCarta, Panther and NCI‐Nature using Enrichr bioinformatics resources (http://amp.pharm.mssm.edu/Enrichr/). A *P*‐value < .05 was considered significant.

### 2.6 miRNA‐T‐PYK expression correlations

We searched for direct expression correlations between pairs of up‐regulated miRNAs and up‐regulated T‐PYKs and pairs of down‐regulated miRNAs and down‐regulated T‐PYKs; and for inverse expression correlations between pairs of up‐regulated miRNAs and down‐regulated T‐PYKs and pairs of down‐regulated miRNAs and up‐regulated T‐PYKs. We built a correlation matrix for deregulated miRNAs with deregulated T‐PYKs. Significance was defined by a *P*‐value from Spearman rank correlation test <.05.

### 2.7 miRNA‐T‐PYK sequence complementarity and common transcription factors

For miRNA‐T‐PYK pairs inversely (negatively) correlated we downloaded and ran RNA22 version 2.0 (https://cm.jefferson.edu/tools-and-downloads/) to identify miRNA binding sites in the T‐PYK sequence.^31^ The threshold used was a *P*‐value (representing the likelihood that the target site loci is random) <.05.

For miRNA‐T‐PYK pairs directly (positively) correlated we retrieved from ENCODE Transcription Factor Targets Dataset the transcription factors (TFs) (from ChIP‐Seq experiments^32^) that are located up to 5000 bp upstream of the transcript of interest. For this step we used the GenomicRanges Bioconductor package from R.^33^ We finally selected the TFs common to the miRNA‐T‐PYK pairs and displayed them using the OmicCircos package from (BioConductor http://bioconductor.org/packages/release/bioc/html/OmicCircos.html).

### 2.8 RNA‐Seq expression across different immune cells

For each gene of interest, we obtained the RNA‐Seq expression across 13 immune cell types and two activated cell types from healthy donors from the Database of Immune Cell Expression, Expression quantitative trait loci (eQTLs) and Epigenomics (DICE) (https://dice-database.org).^34^ Linear RNA‐seq data were downloaded, and we performed further analysis for transcripts which were expressed at >1 average transcripts per million (TPM) across all the different immune cells.

### 2.9 Statistical analysis

Statistical analyses were carried out with the GraphPad Prism 8 software. To determine whether the data followed a normal distribution, the Shapiro‐Wilk normality test was performed. For the comparison between groups *P*‐values were determined with an unpaired *t* test if the data were normally distributed, while the non‐parametric Mann‐Whitney‐Wilcoxon test was applied on data with a non‐normal distribution. All tests were two‐sided, and a *P*‐value < .05 was considered statistically significant.

## 3 RESULTS

### 3.1 The plasma miRNA signature differs between pre‐splenectomy, early post‐splenectomy and late post‐splenectomy

During the time period 2013‐2015, 27 patients (Table S1) were prospectively included in the study and blood was sampled for 25 patients before elective surgery (day D0) and 24 were sampled 7 days after surgery (day D7). Because not all patients returned for follow‐up as the study was designed, we grouped together the patients that were sampled on day 30 and day 90 after surgery (days D30‐90), totalling 28 blood sampled—eleven patients being sampled on both dates. We also grouped the patients sampled on day 180 and 360 (days D180‐360) totalling 11 blood samples, two patients being sampled twice.

**Figure 1:**
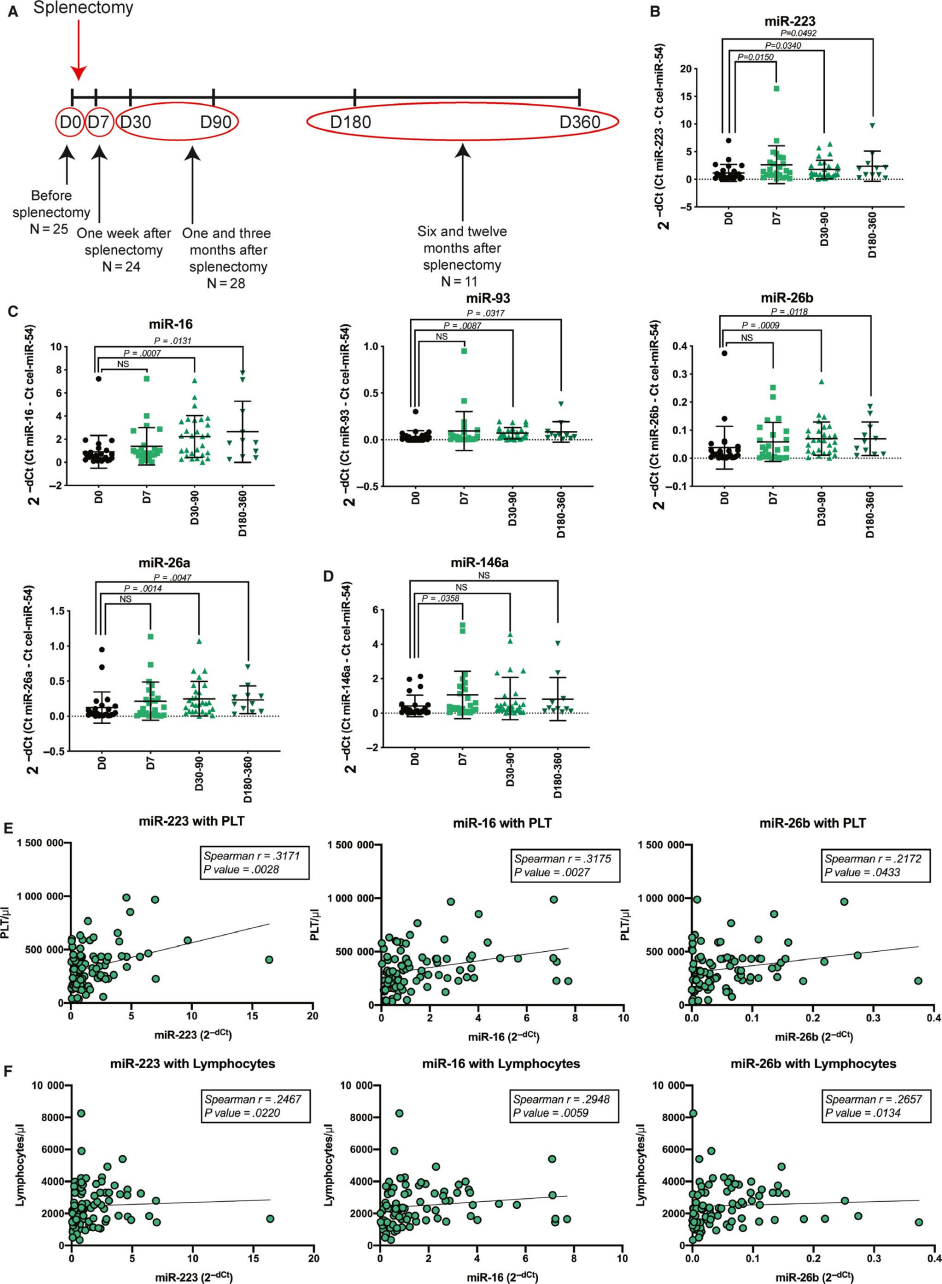
MiRNA expression in plasma before and after splenectomy. A, Design of the study: patients were sampled before splenectomy (D0), 7 d after splenectomy (D7), 30 d after splenectomy (D30), 90 d after splenectomy (D90), 180 d after splenectomy (D180) and 1 y after splenectomy (D360). B, MiR‐223 expression in plasma after splenectomy; C, miR‐16, miR‐93 miR‐26b and miR‐26a expression after splenectomy and D, miR‐146a expression after splenectomy. *P*‐value was calculated using Mann‐Whitney test. Correlation between the expression of miR‐223, miR‐16 and miR‐26b and E, PLT level and F, lymphocytes abundance

The design of the plasma study is presented in Figure 1A. Of all the analysed miRNAs only miR‐223 was statistically up‐regulated early (D7, *P* = .0150) and late (D30‐90, *P* = .034 and D180‐360, *P* = .0492) after splenectomy (Figure 1B). MiR‐223 is well known to be overexpressed in systemic inflammatory response syndrome (SIRS) and down‐regulated in sepsis.^35^ The levels of miR‐16, miR‐93, miR‐26a and miR‐26b did not changed early after splenectomy, but were up‐regulated in the D30‐90 (miR‐16: *P* = .0007; miR‐93: *P* = .0087; miR‐26b: *P* = .0009 and miR‐26a: *P* = .0014) and D180‐360 (miR‐16: *P* = .0131; miR‐93: *P* = .0317; miR‐26b: *P* = .0118 and miR‐26a: *P* = .0047) groups compared to D0 (Figure 1C). Next, we observed that miR‐146a is the only miRNA statistically up‐regulated only immediately after splenectomy (D7, *P* = .0358; Figure 1D). Similarly, to miR‐223, multiple studies reported miR‐146a to be reduced in sepsis compared to SIRS.^35^, ^36^ Very interesting, by excluding the three subtotal/partial splenectomized patients from the analysis, we obtained slightly better statistical results, regarding the six differently expressed miRNAs (Figure S2), but no changes regarding the other miRNA candidates (data not shown), indirectly supporting the idea that the removal of the spleen is the cause of miRNA changes in plasma.

We previously hypothesized that the immunosuppression caused by the removal of the spleen would induce the overexpression of the Kaposi Sarcoma Herpes Virus (KSHV) miRNAs,^5^ similar to what we observed in sepsis.^25^ The expression of the two sepsis specific KSHV‐miRNAs: miR‐k12‐10b and miR‐k12‐12* did not change after splenectomy; moreover, miR‐k12‐12* was undetected in most of the patients before and after splenectomy. The expression level of miR‐486, miR‐342 and miR‐150 was stable between the analysed groups. In addition, miR‐23 showed very low expression levels before and after splenectomy, not being expressed in the plasma of multiple patients (Figure S3A).

In order to assess if the surgical trauma itself is the cause of the miRNA deregulation early after splenectomy we divided our cohort in minimally invasive treated patients (MI, n = 14) and open surgery patients (Open, n = 10). We compared the plasma miRNA expression of the two cohorts 7 days after surgery (D7). None of the 11 miRNA expressed in plasma showed any statistical difference between Open and MI (Figure S3B). Therefore, we concluded that the stress induced by the surgical intervention itself is not the cause of the deregulated miRNA expression.

Finally, we correlated the plasma expression of the six differently expressed miRNAs with the level of total leucocytes, monocytes, lymphocytes, platelets (PLT) and haemoglobin (Hb). The level of miR‐223, miR‐16, miR‐26b, miR‐26a and miR‐146a correlated with the abundance of PLT (Figure 1E and Figure S4A) and the expression of all six miRNAs moderately correlated with the abundance of lymphocytes (Figure 1F and Figure S4B). None of the six miRNAs correlated with the total leucocytes, monocytes or Hb level (data not shown). These results suggest that the deregulated plasma miRNAs could partially originate from PLT and lymphocytes.

### 3.2 Splenectomy induces a reorganization of the non‐coding transcriptome in peripheral blood leucocytes

**Figure 2:**
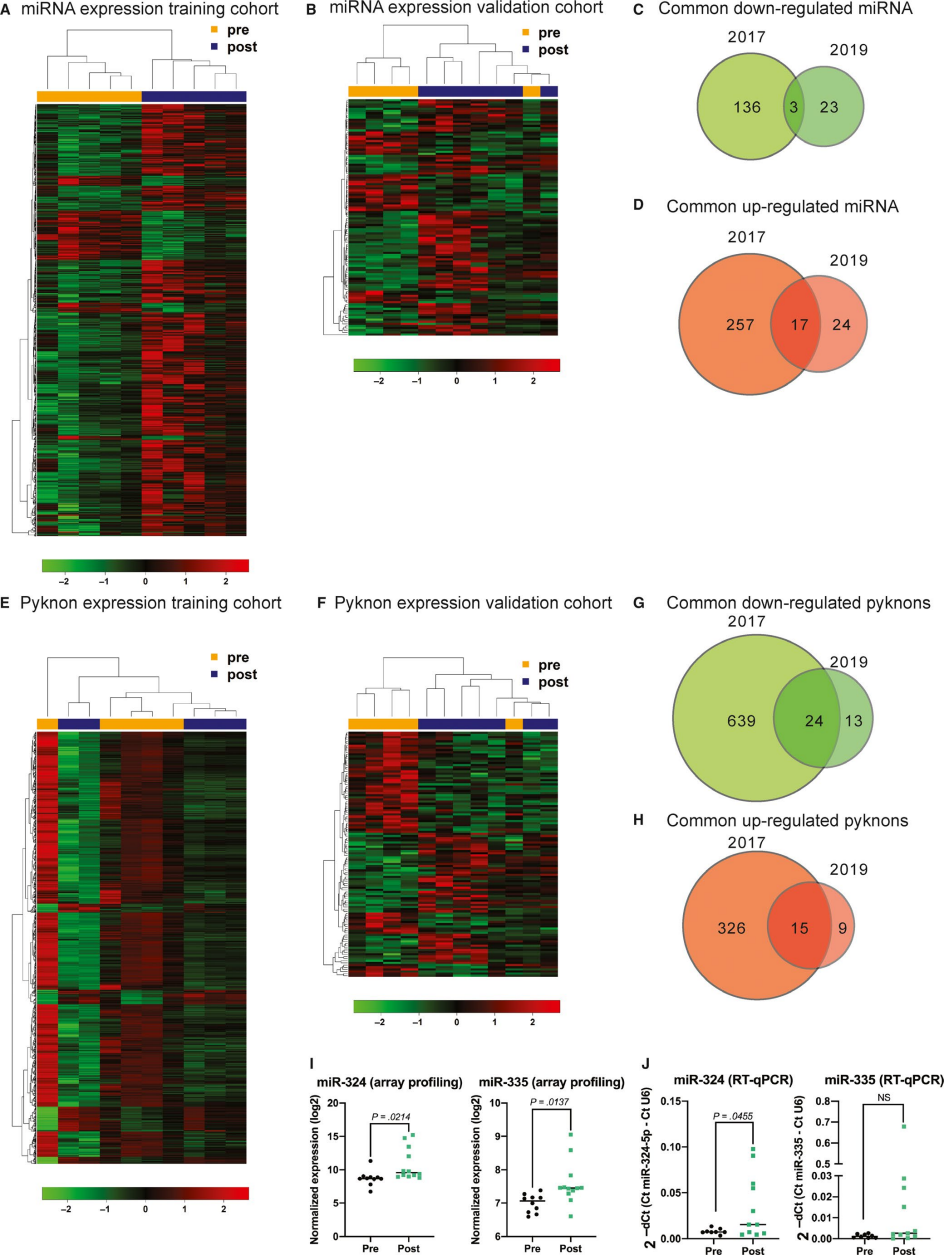
ncRNome array of peripheral blood leucocytes. A, miRNA expression in peripheral blood leucocytes before and after splenectomy in training cohort. B, miRNA expression in peripheral blood leucocytes before and after splenectomy in validation cohort. C, Venn diagram of common down‐regulated miRNA. D, Venn diagram of common up‐regulated miRNAs. E, T‐PYKs expression in peripheral blood leucocytes before and after splenectomy in training cohort. F, T‐PYKs expression in peripheral blood leucocytes before and after splenectomy in validation cohort. G, Venn diagram of common down‐regulated T‐PYKs. H, Venn diagram of common up‐regulated T‐PYKs. I, MiR‐324 and miR‐335 expression in the array profiling and J, miR‐324 and miR‐335 expression using RT‐qPCR. *P*‐value was calculated using unpaired *t* test

In order to explore the changes in ncRNome from circulating blood leucocytes, we performed two independent ncRNA array profiling (2017 training cohort and 2019 validation cohort) on the RNA extracted from peripheral blood leucocytes collected before and 7 to 30 days after splenectomy. In the 2017 training cohort 10 matched samples, from five patients sampled at D0, D7 ‐ three samples and D30 ‐ the other two samples (D7 and D30 were grouped together as post‐splenectomy), were included. Patients' characteristics are listed in Table S2. The patients clustered in pre‐ and post‐splenectomy based on the different miRNA expression. We found 274 up‐regulated and 139 down‐regulated miRNAs (Figure 2A). In the 2019 validation cohort 12 samples from six patients, five samples from D0, five samples from D7 and two samples from D30 (D7 and D30 were grouped together as post‐splenectomy), were included. Patients' characteristics are listed in Table S2. The patients clustered in pre‐ and post‐splenectomy (with one outlier) based on the miRNA expression. We observed 41 up‐regulated and 26 down‐regulated miRNAs (Figure 2B). We detected a clear tendency of having more up‐regulated miRNAs than down‐regulated after splenectomy (Figure S5A and S5B). We looked for common miRNAs between the two cohorts and found 3 common down‐regulated miRNAs (Figure 2C and Table 1) and 17 up‐regulated miRNAs (Figure 2D and Table 1).

**Table 1:** Common up‐ and down‐regulated miRNAs in training and validation cohorts

Name	Direction	FCH 2017	*P*‐value 2017	FCH 2019	*P*‐value 2019
hsa‐mir‐1183	Down	−1.27393	.03113	−1.18910	.03371
hsa‐mir‐4482	Down	−1.20112	.02403	−1.18134	.00547
hsa‐mir‐4776‐1	Down	−1.16736	.03171	−1.19098	.04709
hsa‐mir‐675	Up	2.560167	.03462	3.128122	.00577
hsa‐mir‐324	Up	1.457542	.02040	2.997813	.02767
hsa‐mir‐3685	Up	2.032401	.03613	1.760359	.03325
hsa‐mir‐652	Up	1.956314	.01214	1.697622	.02557
hsa‐mir‐939	Up	1.745731	.01544	1.713752	.04477
hsa‐mir‐409	Up	1.668098	.01710	1.739345	.03260
hsa‐mir‐4326	Up	1.794196	.03021	1.428883	.03194
hsa‐mir‐1257	Up	1.632484	.02338	1.550926	.04808
hsa‐mir‐4436b‐2	Up	1.722482	.01916	1.369651	.02399
hsa‐mir‐4437	Up	1.369138	.02573	1.599208	.01717
hsa‐mir‐4688	Up	1.440179	.02481	1.392915	.03300
hsa‐mir‐335	Up	1.333544	.02235	1.372287	.03255
hsa‐mir‐550b‐1	Up	1.446671	.04414	1.257944	.02142
hsa‐mir‐4652	Up	1.355628	.01477	1.259334	.02772
hsa‐mir‐3610	Up	1.362889	.01384	1.229792	.03763
hsa‐mir‐340	Up	1.236528	.02906	1.158496	.02546
hsa‐mir‐555	Up	1.174054	.04957	1.193683	.03226

Except miRNAs, the other notably deregulated component of the ncRNA transcriptome after splenectomy in peripheral leucocytes were the human/primate‐specific T‐PYKs (Figure S5A and S5B). Pyknons (PYKs) are non‐random patterns of minimum 16 bases in length that repeat at least 40 times in the intergenic portion of the genome.^37^ Many of the PYKs are located in genomic regions which are transcribed (therefore termed T‐PYKs). In the 2017 training cohort the patients clustered relatively well in pre‐ and post‐splenectomy based on the differently expressed T‐PYKs (two post‐splenectomy samples clustered in the pre‐splenectomy group). We found 341 up‐regulated and 663 down‐regulated T‐PYKs after splenectomy (Figure 2E). We validated these findings using the 2019 cohort. The patients were clustered in pre‐ and post‐splenectomy (with one outlier—one pre‐splenectomy patient clustered with the post‐splenectomy ones) based on the differently expressed T‐PYKs. We found 24 up‐regulated and 37 down‐regulated T‐PYKs after splenectomy (Figure 2F). Interestingly, opposite to miRNAs, which showed a tendency to be up‐regulated after splenectomy, more T‐PYKs were down‐regulated after splenectomy (Figure S5A and S5B). Next, we looked for common deregulated T‐PYKs between the 2 cohorts and found 24 common down‐regulated (Figure 2G and Table 2) and 15 up‐regulated T‐PYKs (Figure 2H and Table 2). We checked the genomic location of these T‐PYKs and seven down‐regulated and seven‐up‐regulated were mapping onto introns or exons of known genes (Table 2).

**Table 2:** Common up‐ and down‐regulated T‐PYKs in training and validation cohorts

Name	Direction	FCH 2017	*P*‐value 2017	FCH 2019	*P*‐value 2019	Location	Distance and features
PYK1_r_109421341	Down	−3.157	.01829	−1.499	.03217	*TAF13*	1194
PYK1_r_153234356	Down	−1.580	.03266	−1.476	.04810	*LENEP*	941
PYK10_f_135005869	Down	−2.829	.00914	−1.208	.04615	***PRAP1***	0 (intron)
PYK14_r_99513962	Down	−1.355	.02172	−1.149	.04171	***EVL***	0 (intron)
PYK16_f_14539607	Down	−1.490	.01995	−1.288	.01039	***PARN***	0 (intron)
PYK17_r_18227787	Down	−2.083	.00729	−1.360	.02385	***EVPLL***	0 (intron)
PYK19_f_10908960	Down	−1.253	.01311	−1.2383	.02454	*TIMM29*	7044
PYK19_f_11054965	Down	−2.016	.02183	−1.252	.04332	*LDLR*	5858
PYK19_f_14035273	Down	−1.912	.02366	−1.412	.02138	*PALM3*	4302
PYK19_f_54644916	Down	−3.061	.01896	−1.254	.03844	***PIH1D1***	0 (intron)
PYK19_f_59043367	Down	−1.937	.01422	−1.329	.03617	*lnc‐MYADM‐1*	16 246
PYK19_r_10905877	Down	−2.166	.02455	−1.332	.02717	*TIMM29*	3961
PYK2_f_136313238	Down	−2.490	.01405	−1.211	.04872	*MCM6*	212
PYK20_r_61758560	Down	−1.178	.01054	−1.171	.04750	*RTEL1‐TNFRSF6B*	832
PYK22_f_18663875	Down	−1.528	.02084	−1.258	.03184	*DGCR6L*	17 671
PYK22_f_26666993	Down	−2.197	.00055	−1.192	.04307	***TTC28‐AS1***	0 (intron)
PYK22_r_26390402	Down	−1.219	.04227	−1.254	.02712	*lnc‐CRYBA4‐10*	9451
PYK22_r_41255282	Down	−3.290	.01750	−1.905	.01668	*RRP7A*	9509
PYK3_f_37853846	Down	−2.107	.02052	−1.218	.03221	*ITGA9*	17 561
PYK8_f_141724439	Down	−2.400	.00018	−1.280	.01000	*ERICD*	6726
PYK8_f_23188562	Down	−2.530	.02127	−1.344	.02493	*R3HCC1*	12 779
PYK8_r_24458777	Down	−1.614	.01696	−1.204	.03533	*ADAM7*	18 404
PYK8_r_25973252	Down	−1.598	.03446	−1.639	.04874	***PPP2R2A***	0 (intron)
PYK9_r_124739822	Down	−2.541	.00692	−1.363	.01822	***lnc‐RABGAP1‐1***	0 (exon)
PYK9_f_66967215	Up	2.041	.02159	1.837	.03182	***AQP7P1***	0 (intron)
PYK9_f_121549067	Up	2.630	.02737	1.228	.04395	*lnc‐B3GNT10‐1*	222 843
PYK17_f_18215890	Up	2.377	.00675	1.293	.03002	*EVPLL*	5695
PYK17_f_6818539	Up	1.963	.04250	1.191	.02373	*ALOX12‐AS1*	10 408
PYK8_r_9983280	Up	1.549	.01375	1.574	.04551	***MSRA***	0 (intron)
PYK8_r_26201845	Up	1.785	.00736	1.292	.03956	***PPP2R2A***	0 (intron)
PYK7_r_26015368	Up	1.681	.01638	1.336	.02603	*lnc‐HNRNPA2B1‐2*	48 377
PYK11_f_58077988	Up	1.474	.03587	1.449	.02559	***LPXN***	0 (intron|exon)
PYK22_r_40724325	Up	1.372	.01544	1.388	.04438	*WBP2NL*	135
PYK22_r_40972545	Up	1.272	.03374	1.445	.02897	*OGFRP1*	22 943
PYK19_f_14256192	Up	1.398	.01243	1.178	.02292	*LINC01841*	35 580
PYK9_f_121106135	Up	1.230	.02768	1.329	.02078	***BRINP1***	0 (intron)
PYK4_f_8090605	Up	1.209	.02987	1.330	.02806	***ABLIM2***	0 (intron)
PYK19_f_14462821	Up	1.286	.02914	1.240	.04980	***GIPC1***	0 (intron)
PYK8_r_33519285	Up	1.283	.02138	1.220	.04989	*RNF122*	5314

Gene in bold = the pyknon is located inside the genomic sequence of the gene (intron or exon).

Regarding the other components of the ncRNome, lncRNAs and T‐UCRs, we observed only minimal changes (Figure S5A and S5B). After splenectomy 35 lncRNAs were up‐regulated and 1 down‐regulated according to the 2017 training cohort and 6 lncRNAs up‐regulated and 3 down‐regulated according to the 2019 validation cohort. Of these, none of the down‐regulated lncRNAs were common between cohorts, but 6 lncRNAs were up‐regulated in both cohorts (Figure S5C and S5D and Table S3). T‐UCRs showed only negligible differences, the splenectomy induced the up‐regulation of 63 T‐UCRs and down‐regulation of 3 according to the 2017 training array and 1 T‐UCRs was up‐regulated and 1 down‐regulated according to the 2019 validation array. None of the deregulated T‐UCRs were common between the two arrays (Figure S5E and S5F). Hence, we can conclude that splenectomy induces mostly a rearrangement of the miRNA and T‐PYK component of the non‐coding transcriptome, characterized by opposite tendencies: miRNAs up‐regulation and T‐PYKs down‐regulation.

We used a second method to confirm in the same patient cohort (Table S2), two of the top up‐regulated miRNAs in both array profiling (miR‐324: *P* = .0214 and *miR‐335*: *P* = .0137; Figure 2I). We selected miR‐324 and miR‐335 also because these miRNAs were reported to play functional roles in inflammation and sepsis, respectively.^38^, ^39^ By using RT‐qPCR, we observed that miR‐324 is statistically overexpressed post‐splenectomy in peripheral blood leucocytes (*P* = .0455) and miR‐335 fallowed the same trend like in the array profiling but did not reach statistical significance because of the heterogeneous expression among the patients (Figure 2J).

### 3.3 Deregulated miRNAs and T‐PYKs are part of a complex transcriptional network

**Figure 3:**
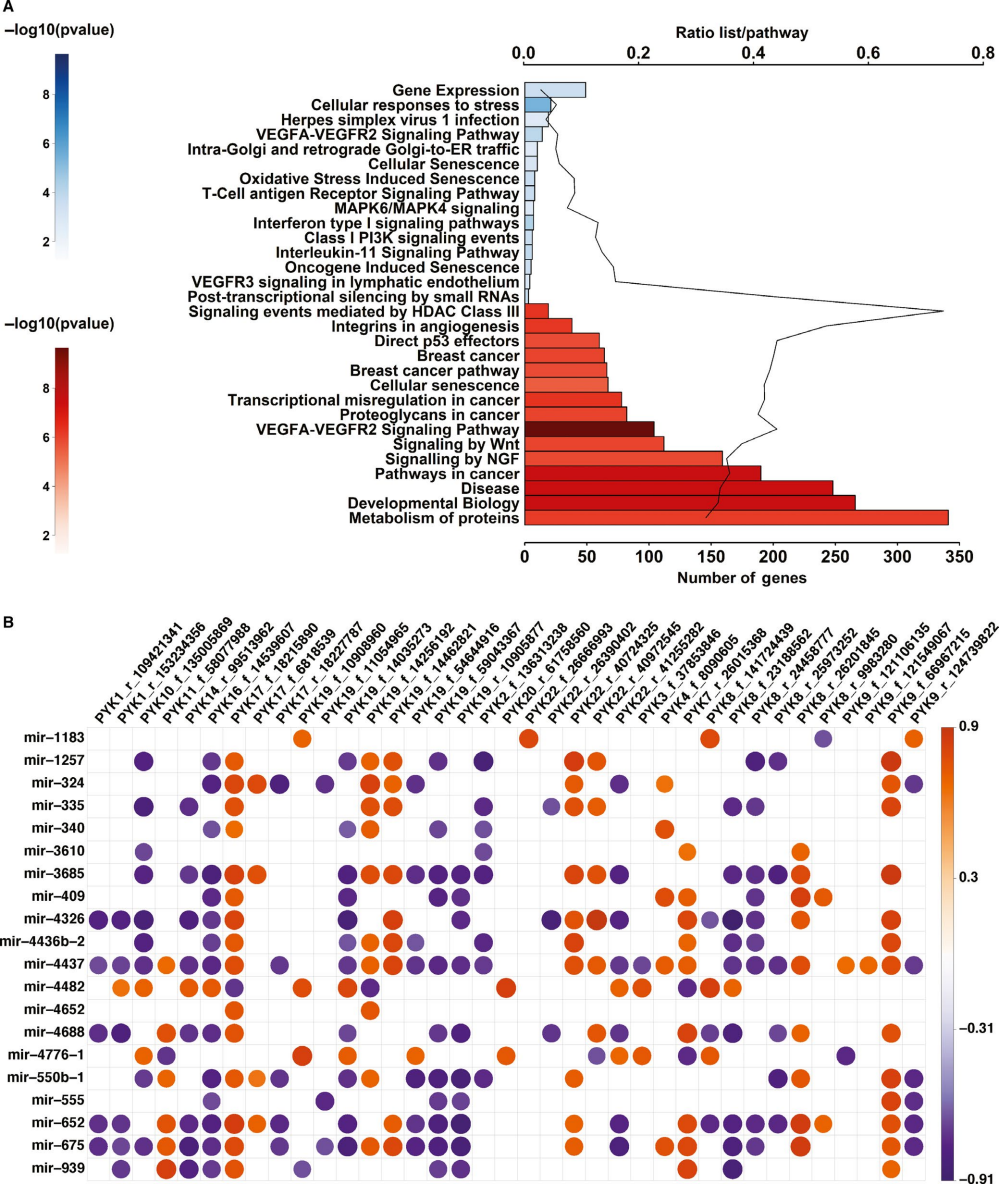
Pathways controlled by the common up‐ and down‐regulated miRNAs. A, Top up‐ and down‐regulated pathways by common miRNAs. Upper *x*‐axis represents ratio of genes targeted by common miRNAs to whole number of genes from pathway. Lower *x*‐axis represents total number of genes controlled by common miRNAs (blue—pathways controlled by down‐regulated miRNAs and red—pathways regulated by up‐regulated miRNAs). B, Corplot library of miRNAs and T‐PYKs correlation, (red—directly correlated pairs and blue—inversely correlated pairs)

To obtain further insights into the mechanism of the deregulated ncRNA transcripts we analysed the downstream pathways controlled by differently expressed cellular miRNAs and checked the expression correlations between deregulated miRNAs and T‐PYKs. We retrieved all the experimentally confirmed targets of the common up‐ and down‐regulated miRNAs from peripheral blood leucocytes (Table S4) and performed pathway analysis. We found 233 up‐regulated pathways and 767 down‐regulated pathways (Table S5); the top 15 down‐ and up‐regulated pathways are presented in Figure 3A. Four of the up‐regulated pathways are linked to the immune response: herpes simplex virus 1 infection, interferon type I signalling pathway, interleukin‐11 (IL‐11) signalling pathway and T‐cell antigen receptor signalling pathway. IL‐11 was proposed recently as a possible new therapy for sepsis.^40^ Many of the top down‐regulated pathways were linked to cancer (p53 effectors, breast cancer, breast cancer pathway, transcriptional misregulation in cancer, proteoglycans in cancer, signalling by Wnt, and pathways in cancer) connecting splenectomy with cancer, a long‐term complication of this surgical procedure.^41^ Curiously, VEGFA‐VEGFR2 pathway was simultaneously up and down‐regulated implying a complex regulation by the differently expressed miRNAs after splenectomy. Moreover, another up‐regulated pathway was VEGFR3 signalling in lymphatic endothelium. The effect of the deregulated miRNAs on VEGFA‐VEGFR pathways can be interpreted in the context of vascular complications associated with splenectomy: myocardial infarction,^42^, ^43^ stroke,^44^ venous thromboembolism^42^, ^45^ and pulmonary hypertension.^42^

Next, we performed miRNA‐T‐PYK expression correlation analysis in both validation and training cohort. We selected only correlations confirmed in both groups and displayed the average of the correlation coefficients using a corrplot library. We detected a high degree of correlation between miRNAs and T‐PYKs, 273 significant correlation pairs of miRNA‐T‐PYKs out of 780 possible (35%) were observed. All miRNAs correlated significantly with multiple common T‐PYKs. Some miRNAs (miR‐4437, miR‐652, miR‐675 and miR‐3685) correlated with twenty ore more T‐PYKs, being central elements of the miRNA‐T‐PYK interaction. Similarly, two T‐PYKs: PYK17_f_18215890 (located 5695 nucleotides upstream of *EVPLL* coding gene) and PYK16_f_14539607 (located in the intron of *PARN* coding gene) correlated with 16 and 15 miRNAs, respectively (Figure 3B). PYK22_f_18663875 (located 17 671 nucleotides upstream of *DGCR6L* coding gene) and PYK8_r_33519285 (located 5314 nucleotides upstream of *RNF122* coding gene) were the only T‐PYKs which did not correlate with any miRNA.

**Figure 4:**
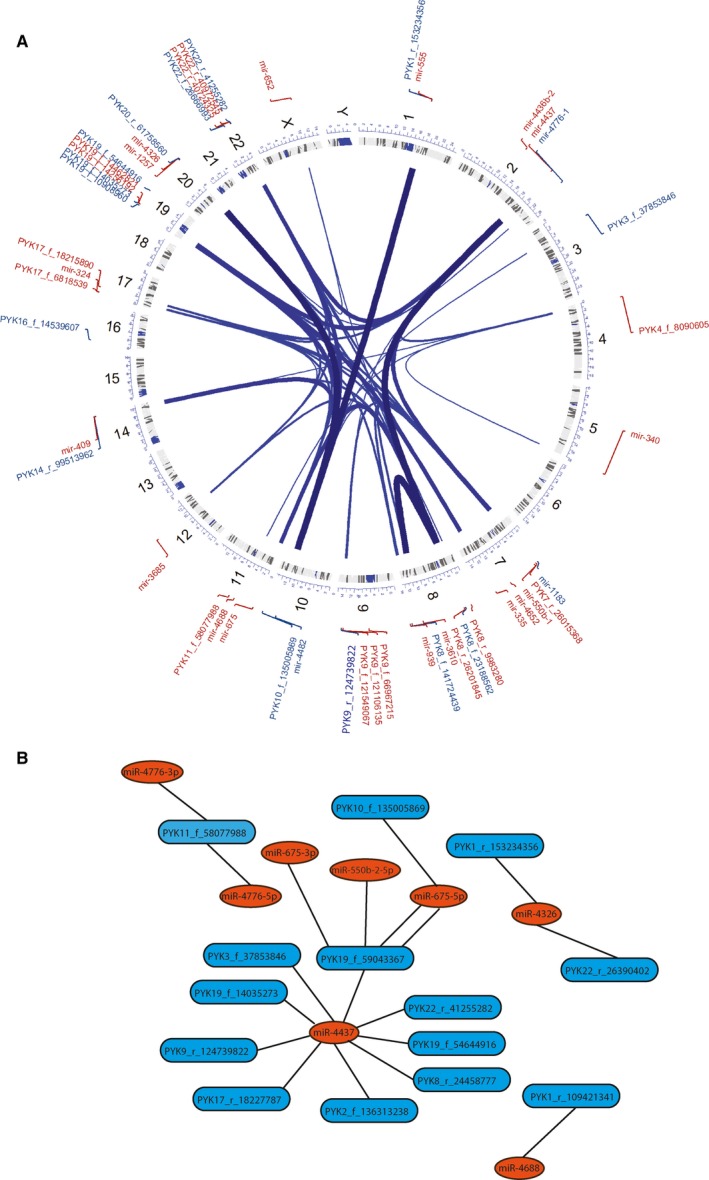
MiRNA‐T‐PYK interaction. A, TF regulation for miRNA‐T‐PYK pairs with same type of change and directly correlated (B) Network of miRNA and T‐PYK pairs inversely correlated and with sequence complementarity

In order to understand the mechanism that controls this synchronized transcription we checked for genomic colocalization of the miRNA‐T‐PYK and only 10 out of 273 (3.66%) pairs shared the same chromosome, but were not closely located (Table S6). We also checked for TFs experimentally confirmed by ChIP‐seq data which are common for miRNA‐T‐PYK pairs that positively correlated. Out of 123 pairs, 62 (50.4%) had experimentally confirmed common TFs, therefore partially explaining the positive correlations between miRNAs and T‐PYKs (Figure 4A and Table S7).

Regarding the miRNA‐T‐PYK pairs, which showed an inverse correlation, we looked for complementary sequences between miRNA and T‐PYK. Complementarity between the transcripts implies a potential direct interaction and could partially explain the inverse correlation.^46^ We found 19 complementary sequences between miRNA and T‐PYK (Figure S6 and Table S8). Two miRNAs showed more than one interaction with a T‐PYK and two T‐PYK were targeted by multiple miRNAs, hence the 19 complementary sequences represented 16 pairs out of the maximum total of 153 (10.26%). These 19 interactions form a complex network that to some extent explains the inverse correlations (Figure 4B).

Overall, these data show that deregulated miRNAs control pathways involved in immunity, cancer and endothelial growth and that a common mechanism exists that controls the expression of miRNAs and T‐PYKs after splenectomy resulting in a complex and highly connected molecular network.

### 3.4 Plasma miRNAs, cellular miRNAs and cellular T‐PYKs have a cell‐specific expression pattern in healthy individuals

**Figure 5:**
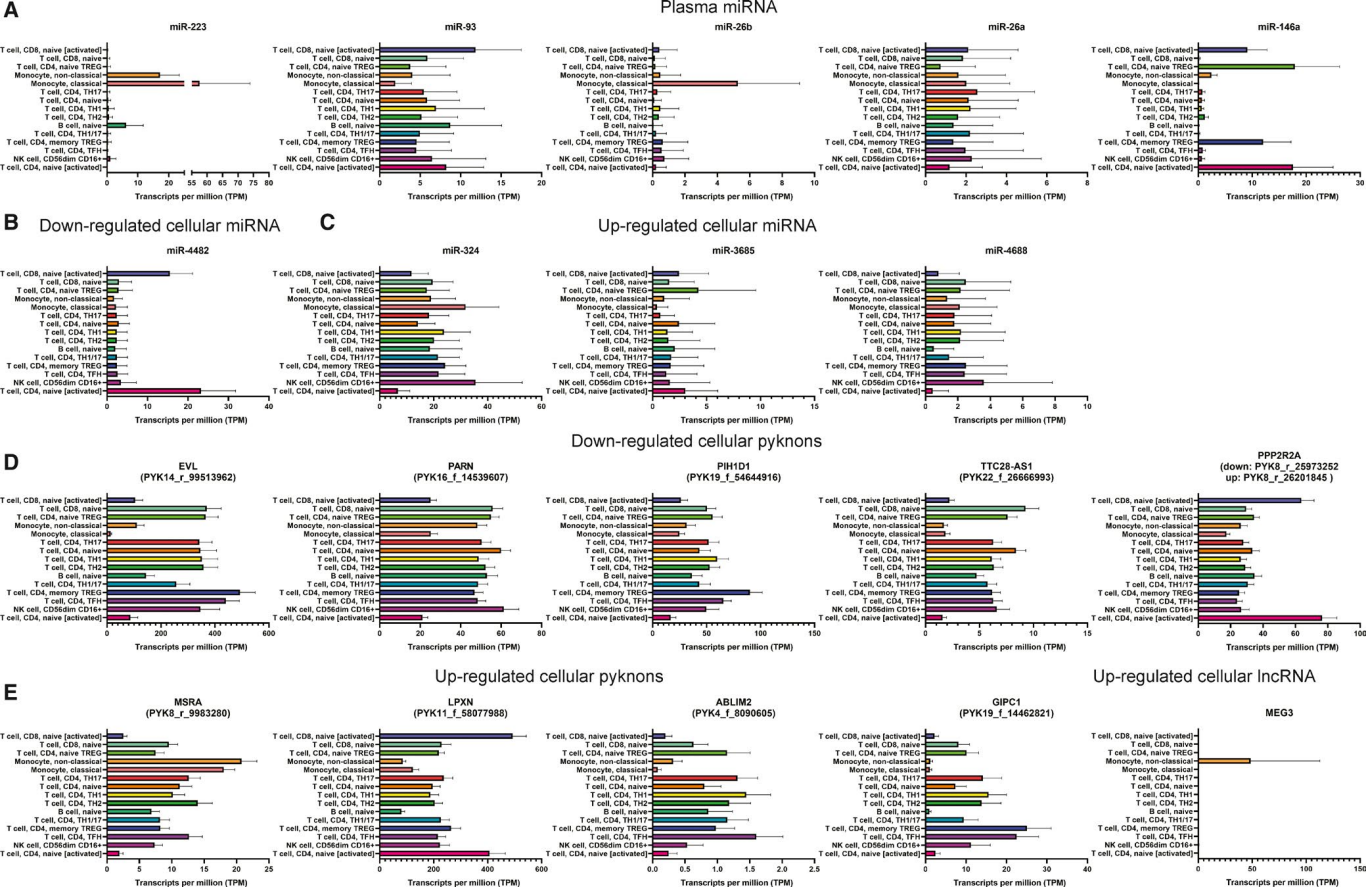
ncRNA expression in 13 immune cell types and 2 activated cell types from 91 healthy donors. A, Plasma miRNA, B, common down‐regulated cellular miRNAs, C, common up‐regulated cellular miRNAs, D, common down‐regulated cellular T‐PYKs, E, common up‐regulated cellular T‐PYKs and F, common up‐regulated lncRNA expression in circulating immune cells

In order to explore the function of these transcribed ncRNAs we assessed their expression in 15 different immune cell types (13 immune cells and 2 activated cell types) from 91 healthy donors from DICE database.^34^ Five out of six plasma miRNAs which we found to be up‐regulated after splenectomy are highly expressed in immune cells of healthy individuals. MiR‐223 showed a very interesting expression pattern, having high expression in classical monocytes (57.53 TPM) and in non‐classical monocyte (17.12 TPM), implying a role in innate immunity. Similarly, miR‐26b showed high expression only in classical monocytes (5.27 TPM), being almost unexpressed in the other subtypes of immune cells. MiR‐146a showed a specific expression in naïve activated CD4 and CD8 positive T cells (17.55 and 9.12 TPM, respectively) and in memory TREG CD4 and CD8 positive T cells (17.94 and 12.04 TPM, respectively), implying a role in regulating the adaptive immune system. Similarly, miR‐93 was highly expressed in naïve activated CD4 and CD8 positive T cells (8.21 and 11.24 TPM, respectively), but also in naïve B cells (8.71 TPM). MiR‐26a showed a moderate expression across all the different cell types, with the highest in T‐helper 17 cells (2.56 TPM; Figure 5A). The only miRNA with very low expression in immune cells of healthy donors was miR‐16, suggesting that it may originate from non‐immune cells.

Only one out of the three cellular miRNAs confirmed in both cohorts to be down‐regulated showed a high expression level in immune cells of healthy donors: miR‐4482. MiR‐4482 is overexpressed in activated CD4 and CD8 positive T cells (23.21 and 15.55 TPM, respectively), implying a role in adaptive immunity (Figure 5B). Of the 17 miRNAs found to be down‐regulated after splenectomy, five miRNAs were expressed in the immune cells of healthy donors. MiR‐324 showed an overall high expression in all the 15 types of immune cells, with the highest in CD16 positive Natural Killer cells (NK) (35.53 TPM) and in non‐classical monocytes (31.93 TPM), implying a role in the innate immune system. MiR‐3685 and miR‐4688 showed a low to medium expression level. MiR‐3685 had the highest expression in CD4 positive TREG T cells (4.25 TPM) and miR‐4688 in NK cells (3.6 TPM) (Figure 5C). MiR‐4326 and miR‐555 showed an average low expression in all the different immune cell lines with no cell‐specific expression pattern (Figure S7).

Next, we checked for the expression of the host genes of the T‐PYKs. From the group of down‐regulated T‐PYKs 8 out of 24 mapped known annotated genes and from the group of up‐regulated T‐PYKs 7 out of 15 mapped known annotated genes (Table 2). Hence, we were able to assess the expression in immune cells only for these T‐PYKs. Five out of eight host genes of the down‐regulated T‐PYKs were high to very high expressed (>100 TPM) in immune cells from healthy individuals. *EVL* (Enah/Vasp‐like) host gene of PYK14_r_99513962 showed a very high expression, especially in memory TREG cells (493 TPM) and in follicular helper T cells (TFH) (441.17 TPM). *EVL* encodes for a protein which is highly expressed in lymph nodes and spleen and was recently linked to transendothelial diapedesis of T cells.^47^ *PARN* (poly(A)‐specific ribonuclease) host gene of PYK16_f_14539607 showed a high expression in most of the immune cells, with the highest in NKs (61.29 TPM). *PIH1D1* (PIH1 domain containing 1) host gene of PYK19_f_54644916 showed a specific high expression in memory TREG cells (90.13 TPM) and the lncRNA *TTC28‐AS1*, the host gene of PYK22_f_26666993 was high in naïve CD4 and CD8 positive T cells (8.37 and 9.37 TPM, respectively). Intriguingly, *PPP2R2A* (protein phosphatase 2 regulatory subunit Balpha) contained simultaneously a down‐ and an up‐regulated T‐PYK: PYK8_r_25973252 and PYK8_r_26201845, proving the fact that transcriptional regulation of T‐PYKs is complex and potentially independent from host genes. *PPP2R2A* showed a highly cell‐specific expression pattern with very high levels in naïve activated CD4 and CD8 positive T cells (76.56 and 63.76 TPM, respectively). The B55α subunit of PPP2R2A was reported as regulator of multiple miRNAs in acute myeloid leukaemia (AML),^48^ partially explaining the synchronized T‐PYK‐miRNA deregulation observed after splenectomy (Figure 5D).

From the group of up‐regulated T‐PYKs, five mapped onto genes expressed in immune cells, including PYK8_r_26201845 which is located on PPP2R2A gene. *MSRA* (methionine sulfoxide reductase A) the host gene of PYK8_r_9983280 was highly expressed in classical and non‐classical monocytes (18.04 and 20.79 TPM, respectively) and recently it has been shown that MSRA protects against LPS induced septic shock.^49^ *LPXN* (leupaxin), the host gene of PYK11_f_58077988, showed a particular expression in immune cells, with very high levels in naïve activated CD4 and CD8 positive T cells (407.03 and 493.3 TPM, respectively). *LPXN* has been reported to form fusion transcripts in AML by translocation and induce leukemogenesis.^50^, ^51^ *ABLIM2* (actin binding LIM protein family member 2) the host gene of PYK4_f_8090605 had an overall low expression level, with the highest expression in TFH cells (1.6 TPM). *GIPC1* (GIPC PDZ domain containing family member 1) the host gene of PYK19_f_14462821 showed a high expression, with the highest levels in CD4 positive TREG cells (25.1 TPM) (Figure 5E).

Finally, we checked the expression of the six common up‐regulated lncRNAs in immune cells. Four out of the six lncRNAs were not available in DICE database, XIST was not expressed and MEG3 showed a highly cell‐specific expression, reaching high levels only in non‐classical monocytes (48.49 TPM) (Figure 5F). MEG3 was recently reported to bind miR‐138 and derepress IL‐1β, intensifying the reaction of macrophages to bacterial infections of the lung and preventing sepsis.^52^

Overall, these findings emphasize that plasma miRNAs, cellular miRNAs and cellular T‐PYKs have a cell‐specific expression pattern and are transcribed in all the different types of immune cells, potentially regulating the complex immune changes observed after splenectomy.

## 4 DISCUSSION

This study is an initial step in understanding the complexity of the infectious and non‐infectious complications that follow splenectomy by studying the ncRNome. Our data prove that splenectomy induces extensive deregulations of miRNAs similar to sepsis in plasma, and a synchronized reorganization of the ncRNome in circulating immune cells.

Firstly, we show that splenectomy is followed by up‐regulation of circulating miRNAs similar to sepsis. MiR‐146a is up‐regulated at 7 days after splenectomy and returns to a basal level at 1 month; miR‐16, miR‐93, miR‐26a and miR‐26b are up‐regulated 1‐3 months after splenectomy and remain up‐regulated at 6‐12 months; and miR‐223 is up‐regulated from day 7 and the high levels persist at 1 year after splenectomy. MiR‐223 is a known modulator of the immune system and was previously reported to be up‐regulated in multiple inflammatory diseases including rheumatoid arthritis, HIV and type 2 diabetes.^30^ Very interesting, Wang et al^35^ showed that serum miR‐223 is up‐regulated in SIRS, but decreases in sepsis, being able to differentiate SIRS from sepsis. Hence, we can envision that the down‐regulation of miR‐223 in splenectomized patients can be a potential marker for the onset of sepsis. In our previous study, we showed that the same group of miRNAs comprising miR‐16, miR‐26a, miR‐26b and miR‐93 are up‐regulated in plasma of non‐surgical septic patients.^25^ Recent data showed that in a murine sepsis model, mice treated with miR‐146a have less apoptotic splenic macrophages and splenectomy diminishes the therapeutic effect of miR‐146a.^53^ This similarity between splenectomy and sepsis could be an initial explanation for the higher risk of sepsis after the removal of the spleen. Close follow‐up of the splenectomized patient can lead to a better understanding of sepsis and to the development of new RNA based therapies. Because splenectomy induces an immunosuppressive state, we hypothesized that it should be followed by an up‐regulation of viral miRNAs, similarly to sepsis.^5^, ^25^ Curiously, the removal of the spleen did not induce any deregulation of the viral miRNAs (miR‐k12‐10b and miR‐k12‐12*), but such data should be followed by an overall viral miRNAs screen.

Secondly, splenectomy induces a major reorganization of the non‐coding transcriptome in peripheral blood leucocytes. In order to understand the intracellular ncRNome we performed a array profiling on the ncRNA from leucocytes from pre‐ and post‐splenectomy patients. The plasma deregulated miRNAs (miR‐223 and miR‐26b) could be found among the miRNAs deregulated in peripheral blood leucocytes by the 2017 array profiling, suggesting that some of the plasma miRNAs could originate from blood leucocytes. Interestingly, we observed a major reorganization of the ncRNome at the cellular level, characterized mainly by miRNA up‐regulation and T‐PYKs down‐regulation. The reorganization of the ncRNome is analogous to the observations made by Xiao et al, who coined the term “genomic storm” after studying the transcriptome (only mRNA) of critically injured humans (including healthy individuals stimulated with endotoxin). The authors observed that over 80% of mRNAs are significantly changed after severe trauma, burn injury or endotoxinemia.^54^ Our array profiling data suggest that splenectomy induces a reprioritization of the non‐coding component of the transcriptome in circulating leucocytes and most probably during sepsis (or other types of systemic inflammation) the amplitude of this deregulation is even higher. The changes we observed at the ncRNA level in peripheral leucocytes could partially explain why splenectomized patients are susceptible to infections and supplementary mechanistic studies are necessary to confirm this. Additionally, our data prove that not only the coding transcriptome should be studied to understand the physiology and pathology of the immune system, but also the non‐coding component, which shows similar degrees of dynamics.

Thirdly, we observed that the cellular deregulated miRNAs control pathways with roles in immunity, endothelial growth and cancer, linking our data with the most common complications of splenectomized population. Moreover, pairwise analysis of deregulated transcripts proved that the expression of miRNAs and T‐PYKs is significantly correlated implying a synchronized deregulation of the ncRNome after splenectomy.

Finally, we assessed the expression of the circulating plasma miRNAs, cellular miRNAs, T‐PYKs and lncRNAs in 13 different types of immune cells and 2 activated cell types. Most of the up‐regulated plasma miRNAs and common cellular non‐coding transcripts are expressed in various immune cells and have highly cell‐specific expression patterns, implying a broad role in immune regulation.

A first limitation of our study is the fact that, except splenectomy, a second intervention carried on all included patients was vaccination against *Haemophilus influenzae*, *Neisseria meningitidis* and *Streptococcus pneumonia* and it is well known that vaccination stimulates the adaptive immune system.^55^ Therefore, it is impossible to determine, using clinical samples, if any of the observed changes in the ncRNA expression are induced by vaccination (adaptive immune system) or by splenectomy (which affects mainly the innate immune system). Additionally, in a recent publication it was observed that the levels of miR‐451a changes after influenza vaccination and anti‐correlates with pro‐inflammatory cytokines.^56^ Interventional in vivo studies are necessary to clarify these details.

A second limitation of the study is the fact that we do not know the precise origin of the circulating miRNAs. It seems that these miRNAs are not originating solely from the circulating immune cells (we did not find the same miRNAs in plasma and circulating immune cells, but we found a positive correlation between the expression of the miRNAs and lymphocytes and PLT, respectively). The spleen coordinates the systemic immunity and splenectomy is followed by changes of multiple types of immune cells^57^ and for instance it is well known that miR‐223 regulates the biology of peritoneal macrophage.^58^ We could speculate that immune cells from other lymphoid and non‐lymphoid organs are also involved in the deregulated plasma miRNA expression, after splenectomy, proving that miRNA transfer is an important cell‐to‐cell communication mechanism.^59^

A third limitation is the heterogeneous and small population (n = 27, circulating miRNA study and n = 11, array profiling) used for this study. We included in this study a population of electively splenectomized patients, most of the patients suffered from benign haematological pathologies or benign pathologies of the spleen, moreover three patients underwent partial/subtotal splenectomy. It would be interesting to check the ncRNome expression in subgroups of patients with specific pathologies and to determine which of the transcripts are specific to a given pathology or to splenectomy per se.

Certainly, such data should be confirmed in additional larger sets of patients analysed by independent groups and further followed by functional studies to understand the specific mechanisms related to splenectomy long‐term complications. Overall, our exploratory study introduces the idea that splenectomy leads to changes at the ncRNA level, which could lead to a better understanding of the infectious and non‐infectious complications that fallow splenectomy.

## CONFLICT OF INTEREST

The authors confirm that there are no conflicts of interest.

## AUTHOR CONTRIBUTIONS

MPD, ST, GAC and CV designed the study. MPD, ST, GAC and CV drafted the manuscript; all authors edited and approved the final version. ST and CV recruited human samples. MPD, KO, ST, MS, DEG performed experiments. MS, MPD, CI, MC, WRH performed the array and computational analysis.

## Data Availability

The array data that support the findings of this study are openly available in GEO at https://www.ncbi.nlm.nih.gov/geo/, accession numbers: GSE133588 for 2017 training cohort and GSE133589 for 2019 validation cohort.

## References

[1] Rubin LG , Schaffner W . Clinical practice. Care of the asplenic patient. N Engl J Med. 2014;371:349‐356. 25054718 10.1056/NEJMcp1314291

[2] Bonnet S , Guédon A , Ribeil J‐A , Suarez F , Tamburini J , Gaujoux S . Indications and outcome of splenectomy in hematologic disease. J Visc Surg. 2017;154:421‐429. 28757383 10.1016/j.jviscsurg.2017.06.011

[3] Davies IL , Cho J , Lewis MH . Splenectomy results from an 18‐year single centre experience. Ann R Coll Surg Engl. 2014;96:147‐150. 24780675 10.1308/003588414X13814021677593 PMC4474245

[4] Dionigi R , Boni L , Rausei S , Rovera F , Dionigi G . History of splenectomy. Int J Surg. 2013;11(Suppl 1):S42‐S43. 24380550 10.1016/S1743-9191(13)60013-8

[5] Dragomir M , Emil Dragoş G , Elena Manga G , A. Călin G , Vasilescu C . Patients After Splenectomy: Old Risks and New Perspectives. Chirurgia (Bucur). 2016;111:393‐399. 27819637 10.21614/chirurgia.111.5.393

[6] Sinwar PD . Overwhelming post splenectomy infection syndrome ‐ review study. Int J Surg. 2014;12:1314‐1316. 25463041 10.1016/j.ijsu.2014.11.005

[7] Weller S , Braun MC , Tan BK , et al. Human blood IgM "memory" B cells are circulating splenic marginal zone B cells harboring a prediversified immunoglobulin repertoire. Blood. 2004;104:3647‐3654. 15191950 10.1182/blood-2004-01-0346 PMC2590648

[8] Kruetzmann S , Rosado MM , Weber H , et al. Human immunoglobulin M memory B cells controlling Streptococcus pneumoniae infections are generated in the spleen. J Exp Med. 2003;197:939‐945. 12682112 10.1084/jem.20022020 PMC2193885

[9] Styrt B . Infection associated with asplenia: risks, mechanisms, and prevention. Am J Med. 1990;88:33N‐42N. 2114797

[10] Tracy ET , Haas KM , Gentry T , et al. Partial splenectomy but not total splenectomy preserves immunoglobulin M memory B cells in mice. J Pediatr Surg. 2011;46:1706‐1710. 21929978 10.1016/j.jpedsurg.2011.04.020

[11] den Haan JM , Kraal G . Innate immune functions of macrophage subpopulations in the spleen. J Innate Immun. 2012;4:437‐445. 22327291 10.1159/000335216 PMC6741446

[12] Serio B , Pezzullo L , Giudice V , et al. OPSI threat in hematological patients. Transl Med UniSa. 2013;6:2‐10. 24251241 PMC3829791

[13] Redis R , Vela L , Lu W , et al. Allele‐specific reprogramming of cancer metabolism by the long non‐coding RNA CCAT2. Mol Cell. 2016;61:520‐534. 26853146 10.1016/j.molcel.2016.01.015 PMC4982398

[14] Calin GA , Liu C‐G , Ferracin M , et al. Ultraconserved regions encoding ncRNAs are altered in human leukemias and carcinomas. Cancer Cell. 2007;12:215‐229. 17785203 10.1016/j.ccr.2007.07.027

[15] Ferdin J , Nishida N , Wu X , et al. HINCUTs in cancer: hypoxia‐induced noncoding ultraconserved transcripts. Cell Death Differ. 2013;20:1675‐1687. 24037088 10.1038/cdd.2013.119 PMC3824588

[16] Munker R , Calin GA . MicroRNA profiling in cancer. Clin Sci (Lond). 2011;121:141‐158. 21526983 10.1042/CS20110005

[17] Dragomir MP , Knutsen E , Calin GA . SnapShot: unconventional miRNA functions. Cell. 2018;174:1038‐e1. 30096304 10.1016/j.cell.2018.07.040

[18] Rigoutsos I , Lee SK , Nam SY , et al. N‐BLR, a primate‐specific non‐coding transcript leads to colorectal cancer invasion and migration. Genome Biol. 2017;18:98. 28535802 10.1186/s13059-017-1224-0 PMC5442648

[19] Dana H , Chalbatani GM , Mahmoodzadeh H , et al. Molecular Mechanisms and Biological Functions of siRNA. Int J Biomed Sci. 2017;13:48‐57. 28824341 PMC5542916

[20] Ozata DM , Gainetdinov I , Zoch A , O’Carroll D , Zamore PD . PIWI‐interacting RNAs: small RNAs with big functions. Nat Rev Genet. 2019;20:89‐108. 30446728 10.1038/s41576-018-0073-3

[21] Mehta A , Baltimore D . MicroRNAs as regulatory elements in immune system logic. Nat Rev Immunol. 2016;16:279‐94. 27121651 10.1038/nri.2016.40

[22] Aune TM , Spurlock CF 3rd . Long non‐coding RNAs in innate and adaptive immunity. Virus Res. 2016;212:146‐60. 26166759 10.1016/j.virusres.2015.07.003 PMC4706828

[23] Giza DE , Fuentes‐Mattei E , Bullock MD , et al. Cellular and viral microRNAs in sepsis: mechanisms of action and clinical applications. Cell Death Differ. 2016;23:1906‐18. 27740627 10.1038/cdd.2016.94 PMC5136497

[24] Vasilescu C , Rossi S , Shimizu M , et al. MicroRNA fingerprints identify miR‐150 as a plasma prognostic marker in patients with sepsis. PLoS ONE. 2009;4:e7405. 19823581 10.1371/journal.pone.0007405 PMC2756627

[25] Tudor S , Giza DE , Lin HY , et al. Cellular and Kaposi's sarcoma‐associated herpes virus microRNAs in sepsis and surgical trauma. Cell Death Dis. 2014;5:e1559. 25476907 10.1038/cddis.2014.515 PMC4649832

[26] Vasilescu C , Dragomir M , Tanase M , et al. Circulating miRNAs in sepsis‐A network under attack: an in‐silico prediction of the potential existence of miRNA sponges in sepsis. PLoS ONE. 2017;12:e0183334. 28820886 10.1371/journal.pone.0183334 PMC5562310

[27] Keshari RS , Silasi R , Popescu NI , et al. Inhibition of complement C5 protects against organ failure and reduces mortality in a baboon model of *Escherichia coli* sepsis. Proc Natl Acad Sci USA. 2017 114(31):E6390‐E6399. 28720697 10.1073/pnas.1706818114 PMC5547645

[28] Silasi R , Keshari RS , Lupu C , et al. Inhibition of contact‐mediated activation of factor XI protects baboons against S aureus‐induced organ damage and death. Blood Adv. 2019;3:658‐69. 30808684 10.1182/bloodadvances.2018029983 PMC6391670

[29] Fuentes‐Mattei E , Giza DE , Shimizu M , et al. Plasma viral miRNAs indicate a high prevalence of occult viral infections. EBioMedicine. 2017;20:182‐92. 28465156 10.1016/j.ebiom.2017.04.018 PMC5478184

[30] Vallee M , Gravel C , Palin MF , et al. Identification of novel and known oocyte‐specific genes using complementary DNA subtraction and microarray analysis in three different species. Biol Reprod. 2005;73:63‐71. 15744023 10.1095/biolreprod.104.037069

[31] Miranda KC , Huynh T , Tay Y , et al. A pattern‐based method for the identification of MicroRNA binding sites and their corresponding heteroduplexes. Cell. 2006;126:1203‐17. 16990141 10.1016/j.cell.2006.07.031

[32] ENCODE Project Consortium . An integrated encyclopedia of DNA elements in the human genome. Nature. 2012;489:57‐74. 22955616 10.1038/nature11247 PMC3439153

[33] Lawrence M , Huber W , Pagès H , et al. Software for computing and annotating genomic ranges. PLoS Comput Biol. 2013;9:e1003118. 23950696 10.1371/journal.pcbi.1003118 PMC3738458

[34] Schmiedel BJ , Singh D , Madrigal A , et al. Impact of genetic polymorphisms on human immune cell gene expression. Cell. 2018;175(1701–15):e16. 10.1016/j.cell.2018.10.022 PMC6289654 30449622

[35] Wang J‐F , Yu M‐L , Yu G , et al. Serum miR‐146a and miR‐223 as potential new biomarkers for sepsis. Biochem Bioph Res Co. 2010;394:184‐8. 10.1016/j.bbrc.2010.02.145 20188071

[36] Wang L , Wang H‐C , Chen C , et al. Differential expression of plasma miR‐146a in sepsis patients compared with non‐sepsis‐SIRS patients. Exp Ther Med. 2013;5:1101‐4. 23596477 10.3892/etm.2013.937 PMC3627686

[37] Rigoutsos I , Huynh T , Miranda K , Tsirigos A , McHardy A , Platt D . Short blocks from the noncoding parts of the human genome have instances within nearly all known genes and relate to biological processes. Proc Natl Acad Sci USA. 2006;103:6605‐10. 16636294 10.1073/pnas.0601688103 PMC1447521

[38] Chen Y , Wang S‐X , Mu R , et al. Dysregulation of the miR‐324‐5p‐CUEDC2 axis leads to macrophage dysfunction and is associated with colon cancer. Cell Rep. 2014;7:1982‐93. 24882011 10.1016/j.celrep.2014.05.007

[39] Gao X‐L , Li J‐Q , Dong Y‐T , et al. Upregulation of microRNA‐335‐5p reduces inflammatory responses by inhibiting FASN through the activation of AMPK/ULK1 signaling pathway in a septic mouse model. Cytokine. 2018;110:466‐78. 29866515 10.1016/j.cyto.2018.05.016

[40] Wan B , Zhang H , Fu H , et al. Recombinant human interleukin‐11 (IL‐11) is a protective factor in severe sepsis with thrombocytopenia: a case‐control study. Cytokine. 2015;76:138‐43. 26276375 10.1016/j.cyto.2015.08.001

[41] Sun L‐M , Chen H‐J , Jeng L‐B , Li T‐C , Wu S‐C , Kao C‐H . Splenectomy and increased subsequent cancer risk: a nationwide population‐based cohort study. Am J Surg. 2015;210:243‐51. 25986002 10.1016/j.amjsurg.2015.01.017

[42] Kristinsson SY , Gridley G , Hoover RN , Check D , Landgren O . Long‐term risks after splenectomy among 8,149 cancer‐free American veterans: a cohort study with up to 27 years follow‐up. Haematologica. 2014;99:392‐8. 24056815 10.3324/haematol.2013.092460 PMC3912973

[43] Schilling RF . Spherocytosis, splenectomy, strokes, and heat attacks. Lancet. 1997;350:1677‐8. 10.1016/s0140-6736(05)64276-6 9400518

[44] Rørholt M , Ghanima W , Farkas DK , Nørgaard M . Risk of cardiovascular events and pulmonary hypertension following splenectomy ‐ a Danish population‐based cohort study from 1996–2012. Haematologica. 2017;102:1333‐41. 28572164 10.3324/haematol.2016.157008 PMC5541868

[45] Boyle S , White RH , Brunson A , Wun T . Splenectomy and the incidence of venous thromboembolism and sepsis in patients with immune thrombocytopenia. Blood. 2013;121:4782‐90. 23637127 10.1182/blood-2012-12-467068 PMC3674676

[46] Bartel DP . MicroRNAs: target recognition and regulatory functions. Cell. 2009;136:215‐33. 19167326 10.1016/j.cell.2009.01.002 PMC3794896

[47] Estin ML , Thompson SB , Traxinger B , Fisher MH , Friedman RS , Jacobelli J . Ena/VASP proteins regulate activated T‐cell trafficking by promoting diapedesis during transendothelial migration. Proc Natl Acad Sci USA. 2017;114:E2901‐E10. 28320969 10.1073/pnas.1701886114 PMC5389297

[48] Ruvolo PP , Ruvolo VR , Jacamo R , et al. The protein phosphatase 2A regulatory subunit B55alpha is a modulator of signaling and microRNA expression in acute myeloid leukemia cells. Biochim Biophys Acta. 2014;1843:1969‐77. 24858343 10.1016/j.bbamcr.2014.05.006 PMC4165504

[49] Singh MP , Kim KY , Kwak G‐H , Baek S‐H , Kim H‐Y . Methionine sulfoxide reductase A protects against lipopolysaccharide‐induced septic shock via negative regulation of the proinflammatory responses. Arch Biochem Biophys. 2017;631:42‐8. 28803836 10.1016/j.abb.2017.08.008

[50] Dai HP , Xue YQ , Zhou JW , et al. LPXN, a Member of the Paxillin Superfamily, Is Fused to RUNX1 in an Acute Myeloid Leukemia Patient with a t(11;21)(q12;q22) Translocation. Gene Chromosome Canc. 2009;48:1027‐36. 10.1002/gcc.20704 19760607

[51] Abe A , Yamamoto Y , Iba S , et al. ETV6‐LPXN fusion transcript generated by t(11;12)(q12.1;p13) in a patient with relapsing acute myeloid leukemia with NUP98‐HOXA9. Gene Chromosome Canc. 2016;55:242‐50. 10.1002/gcc.22327 26542893

[52] Li R , Fang L , Pu Q , et al. MEG3‐4 is a miRNA decoy that regulates IL‐1beta abundance to initiate and then limit inflammation to prevent sepsis during lung infection. Sci Signal. 2018;11(536):eaao2387. 29945883 10.1126/scisignal.aao2387 PMC6637737

[53] Funahashi Y , Kato N , Masuda T , et al. miR‐146a targeted to splenic macrophages prevents sepsis‐induced multiple organ injury. Lab Invest. 2019;99:1130‐42. 30700845 10.1038/s41374-019-0190-4

[54] Xiao W , Mindrinos MN , Seok J , et al. A genomic storm in critically injured humans. J Exp Med. 2011;208:2581‐90. 22110166 10.1084/jem.20111354 PMC3244029

[55] Slifka MK , Amanna I . How advances in immunology provide insight into improving vaccine efficacy. Vaccine. 2014;32:2948‐57. 24709587 10.1016/j.vaccine.2014.03.078 PMC4096845

[56] Miyashita Y , Ishikawa K , Fukushima Y , Kouwaki T , Nakamura K , Oshiumi H . Immune‐regulatory microRNA expression levels within circulating extracellular vesicles correspond with the appearance of local symptoms after seasonal flu vaccination. PLoS ONE. 2019;14:e0219510. 31287847 10.1371/journal.pone.0219510 PMC6615615

[57] Bronte V , Pittet MJ . The spleen in local and systemic regulation of immunity. Immunity. 2013;39:806‐18. 24238338 10.1016/j.immuni.2013.10.010 PMC3912742

[58] Zhang N , Fu L , Bu Y , Yao Y , Wang Y . Downregulated expression of miR‐223 promotes Toll‐like receptor‐activated inflammatory responses in macrophages by targeting RhoB. Mol Immunol. 2017;91:42‐8. 28881218 10.1016/j.molimm.2017.08.026

[59] Redis RS , Calin S , Yang Y , You MJ , Calin GA . Cell‐to‐cell miRNA transfer: from body homeostasis to therapy. Pharmacol Ther. 2012;136:169‐74. 22903157 10.1016/j.pharmthera.2012.08.003 PMC3855335

