# Supplementary material for: The non‐coding RNome after splenectomy
Source: J Cell Mol Med. 2019 Sep 8;23(11):7844–58. doi: 10.1111/jcmm.14664 (PMC6815812; doi:10.1111/jcmm.14664)
Supplement: Supplementary file 1 [file JCMM-23-7844-s001.pdf]

Supporting Information

The non-coding RNome after splenectomy

Mihnea P. Dragomir[1,2,#], Stefan Tudor[2,#], Keishi Okubo[1], Masayoshi Shimizu[1], Meng Chen[1], Dana Elena Giza[3], William Ruixian He[1], Cristina Ivan[1,4], George A. Calin[1,4,] Catalin Vasilescu[2,*]*

Supplementary Figure 1

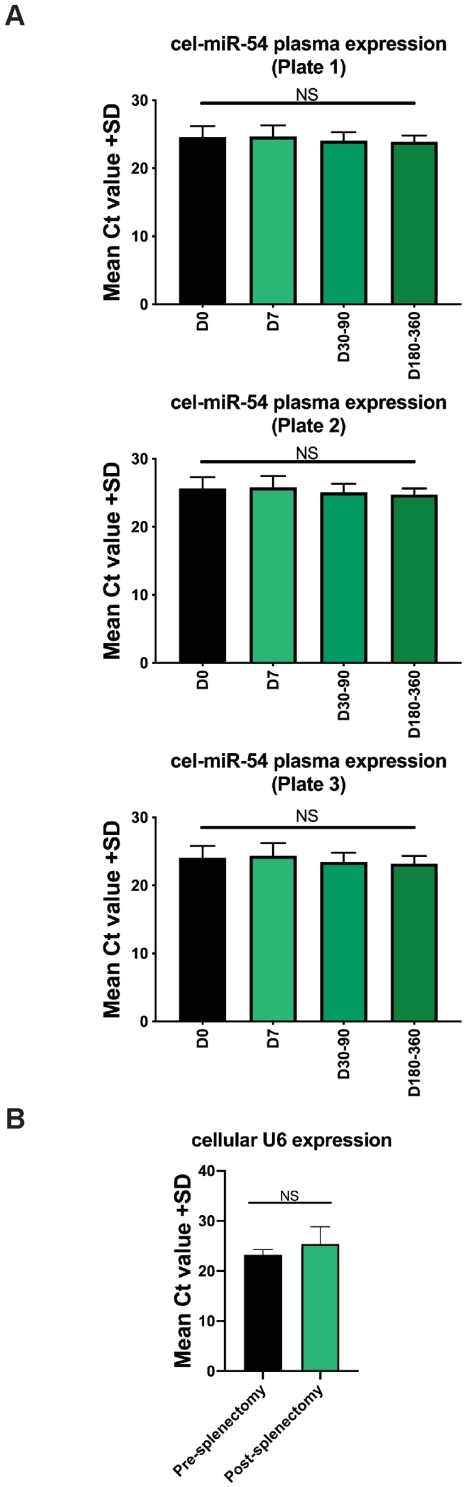

Supplementary Figure S1. (**A**) Normalizer mean Ct value and SD between the analyzed subgroups for plasma study. Plate 1 contained miR-150, miR-16, miR-93 and miR-k12-10b (P=0.2475); Plate 2 contained miR-146a, miR-26a, miR-26b, miR-223 and miR-486 (P=0.1239); Plate 3 contained miR-23, miR-342 and miR-182 (P=0.1080). P-value was calculated using ordinary one-way ANOVA. (**B**) Normalizer mean Ct value and SD between the analyzed subgroups for circulating peripheral blood leukocytes study (P=0.1090). P-value was calculated using unpaired t-test.

Supplementary Figure 2

Only total splenectomy samples

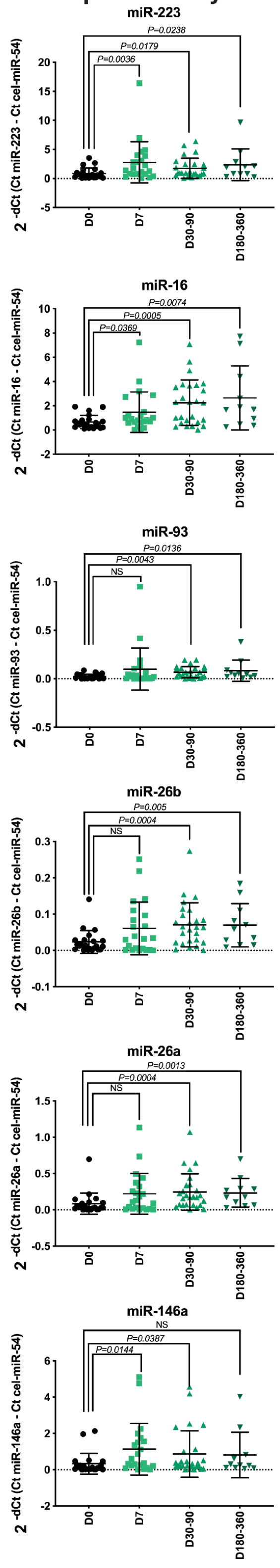

Supplementary Figure S2. MiRNA expression in plasma before and after splenectomy after excluding the patients who underwent partial or subtotal splenectomy. P-value was calculated using unpaired t-test.

Supplementary Figure 3

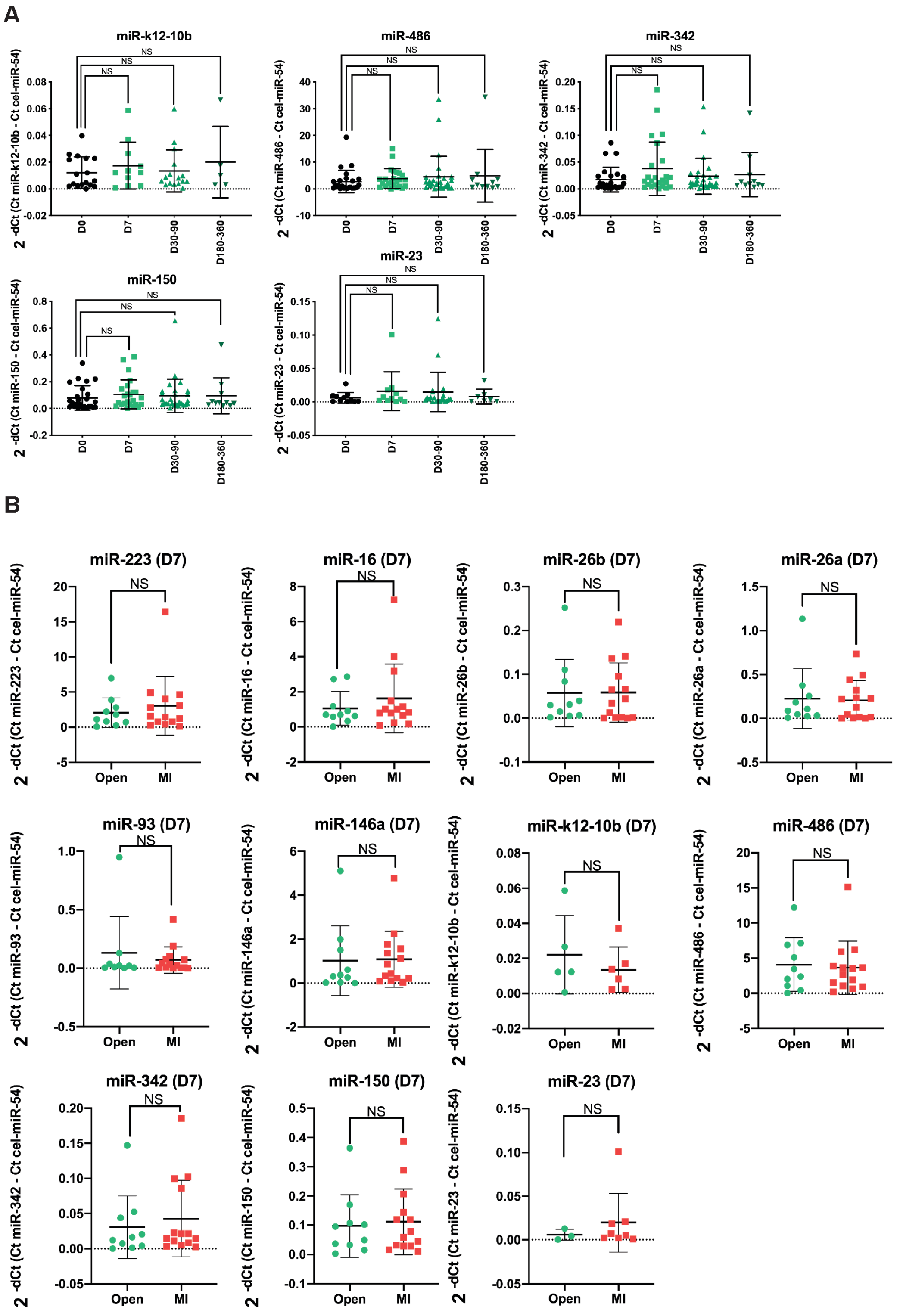

Supplementary Figure S3. (**A**) Not significantly changed miRNA in plasma before and after splenectomy and (**B**) miRNA level in open vs MI surgery at D7. NS – not statistic, MI – minimally invasive. P-value was calculated using ordinary student t-test.

Supplementary Figure 4

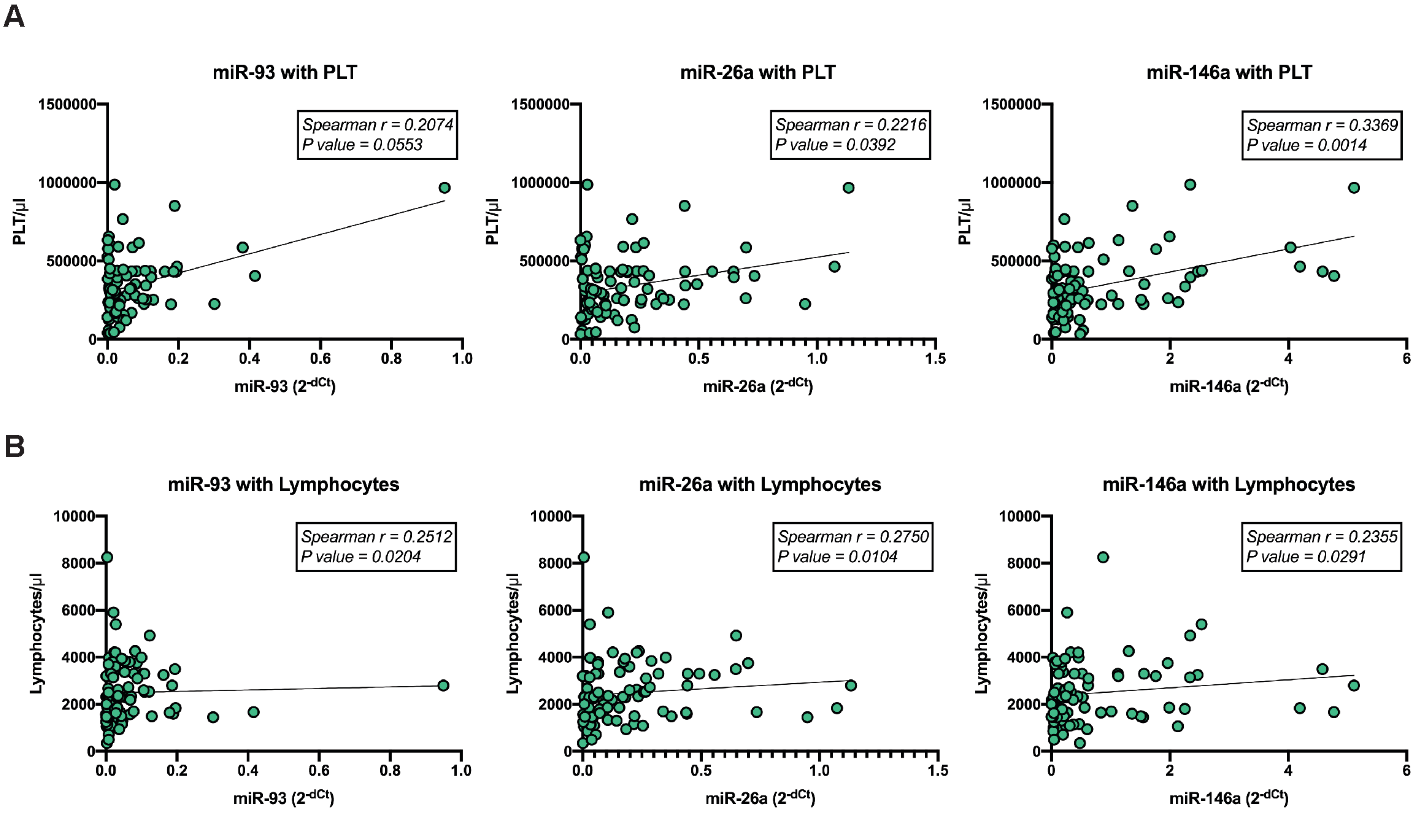

Supplementary Figure S4. Correlation between the expression of miR-93, miR-26a and miR-146a and (**A**) PLT level and (**B**) lymphocytes abundance.

Supplementary Figure 5

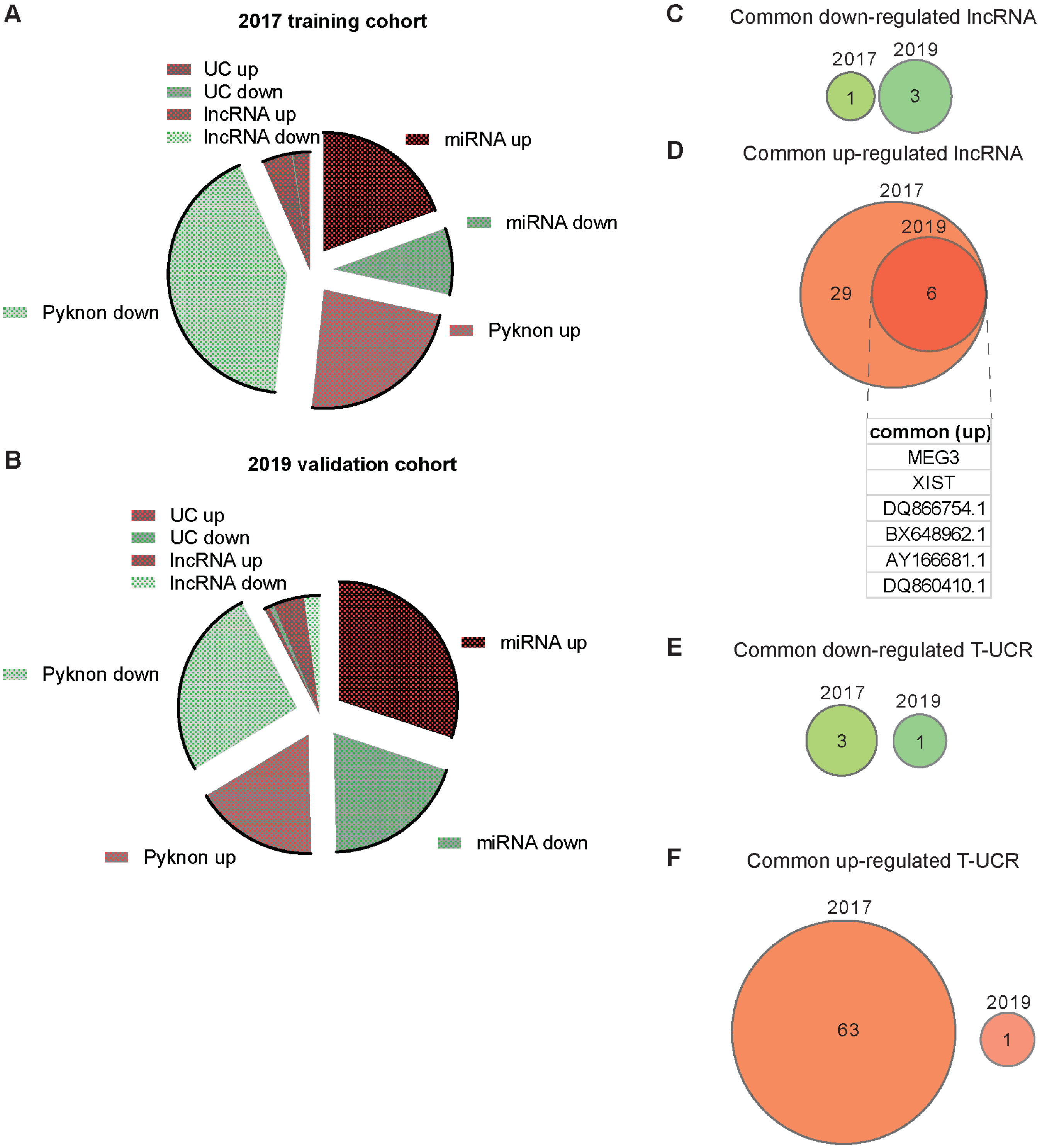

Supplementary Figure S5. Distribution of the ncRNA transcriptome after splenectomy in (**A**) 2017 training cohort and (**B**) **2019** validation cohort. Venn diagram of (**C**) common down-regulated lncRNAs, (**D**) common up-regulated lncRNAs, (**E**) common down-regulated T-UCR, and (**F**) common up-regulated T-UCR.

Supplementary Figure 6

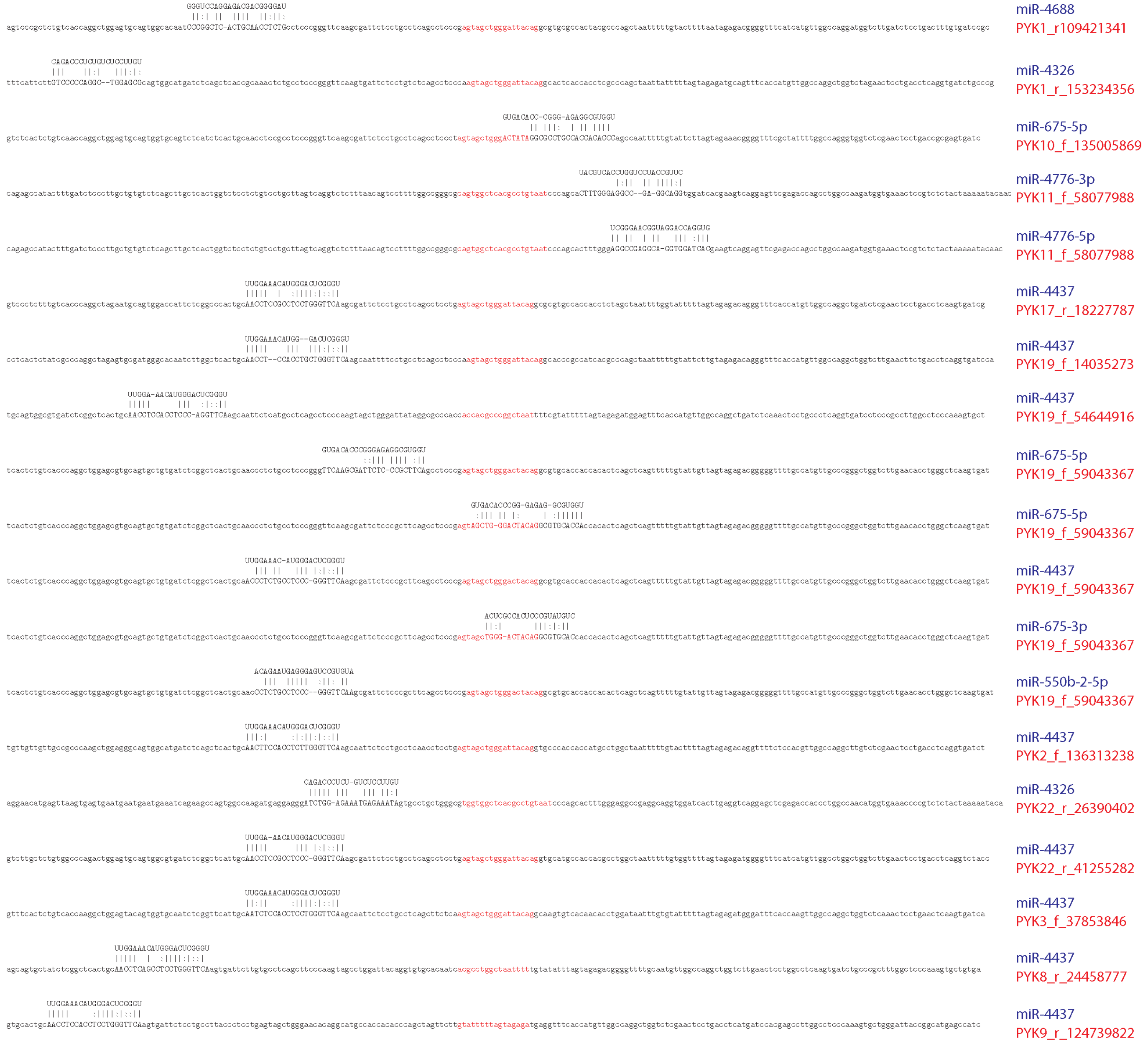

Supplementary Figure S6. Complementarity between miRNA and T-PYK pairs which show inverse correlation.

Supplementary Figure 7

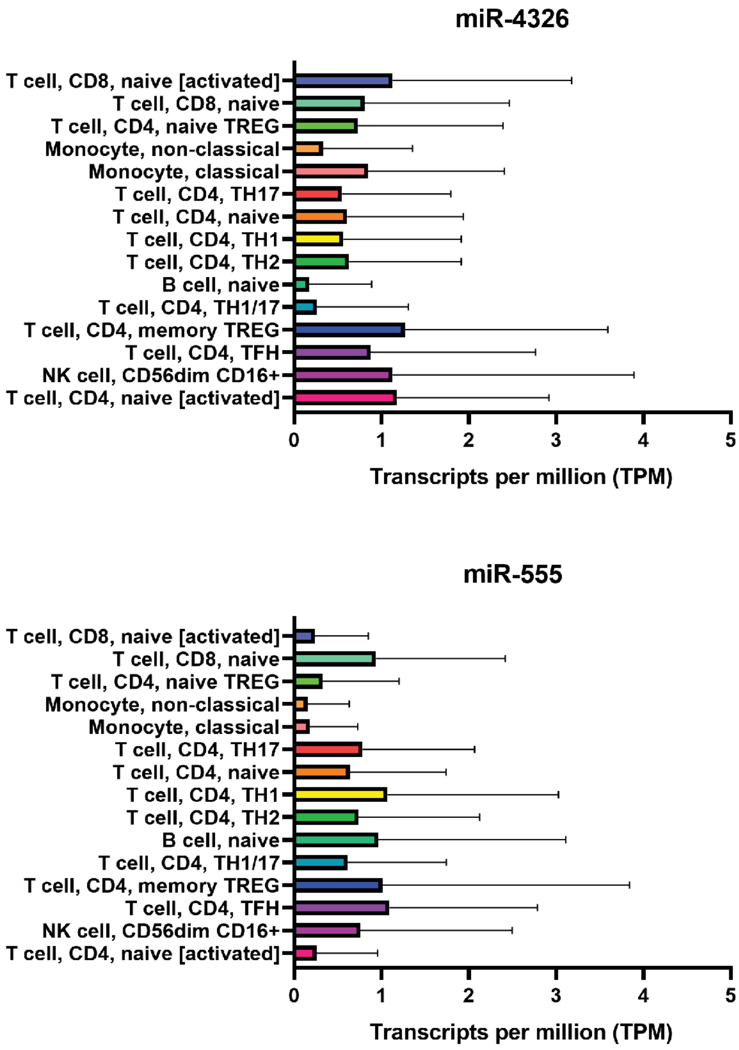

Supplementary Figure S7. MiR-4326 and miR-555 expression in 13 immune cell types and 2 activated cell types from 91 healthy donors.

Supplementary Table S1. Demographic, clinical and operative characteristics of included patients in plasma study (n=27).

**Characteristics**	**Splenectomized patients** n (%)
**Age groups**	
0-19 years	5 (18.52)
20-39 years	8 (29.63)
40-59 years	7 (25.93)
>59 years	7 (25.93)
**Gender**	
Female	15 (55.56)
Male	12 (44.44)
**Surgical indication**	
Immune thrombocytopenic purpura	12 (44.44)
Non-parasitic splenic cyst	3 (11.11)
Spherocytosis	3 (11.11)
Splenomegaly in cirrhotic patient	3 (11.11)
Splenomegaly of no known origin	2 (7.41)
Autoimmune hemolytic anemia	1 (3.7)
Hydatid cyst	1 (3.7)
Hematologic malignancy	1 (3.7)
Hemangioma	1 (3.7)
**Surgical approach**	
Open	10 (37.04)
Minimally invasive (laparoscopic/robotic)	17 (62.96)
**Type of procedure**	
Total splenectomy	24 (88.89)
Subtotal (<15%) or partial splenectomy (> 20-30%)	3 (11.11)

Supplementary Table S2. Demographic, clinical, operative characteristics of included patients in circulating peripheral blood leukocytes study (n=11).

**Patient number**	**Patient characteristics**					**Time point of samples included in array**		
	**Age**	**Gender**	**Indication**	**Procedure**	**Approach**	**Day 0**	**Day 7**	**Day 30**
**2017 training cohort**								
**1**	45	Female	Immune thrombocytopenic purpura	Total	Laparoscopic	yes	no	yes
**2**	65	Female	Hydatid cyst	Total	Open	yes	yes	no
**3**	16	Female	Spherocytosis	Subtotal	Robotic	yes	no	yes
**4**	53	Female	Immune thrombocytopenic purpura	Total	Laparoscopic	yes	yes	no
**5**	10	Female	Spherocytosis	Subtotal	Robotic	yes	yes	no
**2019 validation cohort**								
**6**	30	Male	Immune thrombocytopenic purpura	Total	Laparoscopic	yes	yes	yes
**7**	50	Male	Immune thrombocytopenic purpura	Total	Laparoscopic	yes	yes	no
**8**	42	Female	Immune thrombocytopenic purpura	Total	Laparoscopic	yes	yes	no
**9**	28	Female	Spherocytosis	Total	Robotic	yes	yes	no
**10**	13	Male	Thalassemia major	Total	Laparoscopic	yes	yes	no
**11**	36	Female	Splenomegaly of no known origin	Total	Open	no	no	yes

Supplementary Table S3. Common up-regulated lncRNA in training and validation cohorts.

Name (Symbol official)	Direction	FCH 2017	P value_2017	FCH 2019	P value_2019
gi|381140035|ref|NR_003531.3| Homo sapiens maternally expressed 3 (non-protein coding) (**MEG3**), transcript variant 3, long non-coding RNA	up	1.63067	0.01057	2.04395	0.02830
gi|383087740|ref|NR_001564.2| Homo sapiens X inactive specific transcript (non-protein coding) (**XIST**), long non-coding RNA	up	1.85138	0.03707	1.38496	0.01381
gi|117422529|gb|**DQ866754.1**| Homo sapiens intronic SAP18 antisense RNA sequence	up	1.60067	0.01123	1.33674	0.00447
gi|34368134|emb|**BX648962.1**| Homo sapiens mRNA; cDNA DKFZp686K1684 (from clone DKFZp686K1684)	up	1.39101	0.02045	1.48328	0.02160
gi|25361251|gb|**AY166681.1**| Homo sapiens non-small cell lung carcinoma noncoding RNA, partial sequence	up	1.28793	0.01816	1.31515	0.04584
gi|113129070|gb|**DQ860410.1**| Homo sapiens human accelerated region 1 reverse a mRNA, complete sequence	up	1.29558	0.03161	1.16935	0.04664

Supplementary Table S4. Experimentally confirmed targets of the cellular up- and down-regulated miRNAs retrieved from miRTarBase (excel file).

Supplementary Table S5. Up- and down-regulated pathways of cellular miRNAs retrieved from Enrichr (excel file).

Supplementary Table S6. 273 significantly correlated miRNA-T-PYK pairs and their genomic location in hg19 (excel file).

Supplementary Table S7. Directly correlated miRNA-T-PYK pairs and their common Transcription Factors (excel file).

Supplementary Table S8. Inversely correlated miRNA-T-PYK pairs that show sequence complementarity according to RNA22 (https://cm.jefferson.edu/data-tools-downloads/rna22-full-sets-of-predictions/)

miRNA	T-PYK	Starting position	miRNA sequence	T-PYK interaction sequence	P value
miR-4326	PYK1_r_153234356	11	CAGACCCTCTGTCTCCTTGT	GTCCCCCAGGC--TGGAGCG	0.0288
miR-4326	PYK22_r_26390402	67	CAGACCCTCT-GTCTCCTTGT	ATCTGG-AGAAATGAGAAATA	0.048
miR-4437	PYK19_f_59043367	54	TTGGAAAC-ATGGGACTCGGGT	ACCCTCTGCCTCCC-GGGTTCA	0.0192
miR-4437	PYK22_r_41255282	54	TTGGA-AACATGGGACTCGGGT	AACCTCCGCCTCCC-GGGTTCA	0.0393
miR-4437	PYK19_f_14035273	54	TTGGAAACATGG--GACTCGGGT	AACCT--CCACCTGCTGGGTTCA	0.0327
miR-4437	PYK9_r_124739822	10	TTGGAAACATGGGACTCGGGT	AACCTCCACCTCCTGGGTTCA	0.00174
miR-4437	PYK19_f_54644916	28	TTGGA-AACATGGGACTCGGGT	AACCTCCACCTCCC-AGGTTCA	0.0009
miR-4437	PYK17_r_18227787	54	TTGGAAACATGGGACTCGGGT	AACCTCCGCCTCCTGGGTTCA	0.0029
miR-4437	PYK2_f_136313238	54	TTGGAAACATGGGACTCGGGT	AACTTCCACCTCTTGGGTTCA	0.0156
miR-4437	PYK3_f_37853846	54	TTGGAAACATGGGACTCGGGT	AATCTCCACCTCCTGGGTTCA	0.00045
miR-4437	PYK8_r_24458777	25	TTGGAAACATGGGACTCGGGT	AACCTCAGCCTCCTGGGTTCA	0.00029
miR-4688	PYK1_r_109421341	41	GGGTCCAGGAGACGACGGGGAT	CCCGGCTC-ACTGCAACCTCTG	0.0224
miR-4776-3p	PYK11_f_58077988	128	TACGTCACCTGGTCCTACCGTTC	CTTTGGGAGGCC--GA-GGCAGG	0.0372
miR-4776-5p	PYK11_f_58077988	135	TCGGGAACGGTAGGACCAGGTG	AGGCCGAGGCA-GGTGGATCAC	0.0372
miR-550b-2-5p	PYK19_f_59043367	56	ACAGAATGAGGGAGTCCGTGTA	CCTCTGCCTCCC--GGGTTCAA	0.0192
miR-675-3p	PYK19_f_59043367	107	ACTCGCCACTCCCGTATGTC	TGGG-ACTACAGGCGTGCAC	0.0192
miR-675-5p	PYK19_f_59043367	71	GTGACACCCGGGAGAGGCGTGGT	TTCAAGCGATTCTC-CCGCTTCA	0.0192
miR-675-5p	PYK19_f_59043367	104	GTGACACCCGG-GAGAG-GCGTGGT	AGCTG-GGACTACAGGCGTGCACCA	0.0192
miR-675-5p	PYK10_f_135005869	111	GTGACACC-CGGG-AGAGGCGTGGT	ACTATAGGCGCCTGCCACCACACCC	0.0416
